# Isoforms of Melanopsin Mediate Different Behavioral Responses to Light

**DOI:** 10.1016/j.cub.2015.07.071

**Published:** 2015-09-21

**Authors:** Aarti Jagannath, Steven Hughes, Amr Abdelgany, Carina A. Pothecary, Simona Di Pretoro, Susana S. Pires, Athanasios Vachtsevanos, Violetta Pilorz, Laurence A. Brown, Markus Hossbach, Robert E. MacLaren, Stephanie Halford, Silvia Gatti, Mark W. Hankins, Matthew J.A. Wood, Russell G. Foster, Stuart N. Peirson

**Affiliations:** 1 Nuffield Laboratory of Ophthalmology, John Radcliffe Hospital, University of Oxford, Levels 5-6 West Wing, Headley Way, Oxford OX3 9DU, UK; 2 F. Hoffmann-La Roche AG, Pharma Research and Early Development, DTA Neuroscience pRED, Grenzacherstrasse 124, Basel 4070, Switzerland; 3 Department of Physiology, Anatomy and Genetics, South Parks Road, Oxford OX1 3QX, UK; 4 Axolabs GmbH, Fritz-Hornschuch-Straße 9, 95326 Kulmbach, Germany

## Abstract

Melanopsin (OPN4) is a retinal photopigment that mediates a wide range of non-image-forming (NIF) responses to light [1, 2] including circadian entrainment [3], sleep induction [4], the pupillary light response (PLR) [5], and negative masking of locomotor behavior (the acute suppression of activity in response to light) [6]. How these diverse NIF responses can all be mediated by a single photopigment has remained a mystery. We reasoned that the alternative splicing of melanopsin could provide the basis for functionally distinct photopigments arising from a single gene. The murine melanopsin gene is indeed alternatively spliced, producing two distinct isoforms, a short (OPN4S) and a long (OPN4L) isoform, which differ only in their C terminus tails [7]. Significantly, both isoforms form fully functional photopigments [7]. Here, we show that different isoforms of OPN4 mediate different behavioral responses to light. By using RNAi-mediated silencing of each isoform in vivo, we demonstrated that the short isoform (OPN4S) mediates light-induced pupillary constriction, the long isoform (OPN4L) regulates negative masking, and both isoforms contribute to phase-shifting circadian rhythms of locomotor behavior and light-mediated sleep induction. These findings demonstrate that splice variants of a single receptor gene can regulate strikingly different behaviors.

## Highlights

• The retinal photopigment melanopsin is alternatively spliced
• The isoforms mediate different physiological and behavioral responses to light
• The short variant regulates pupil size, the long, negative masking of activity
• Both variants regulate sleep and phase shifting of circadian rhythms

## Teaser

We show that the splice variants of the retinal photopigment melanopsin regulate different non-image-forming responses to light, including, for example, pupil constriction, which is mediated by the short variant alone. Our findings demonstrate that splice variants of a single receptor gene can regulate strikingly different behaviors.

## Results and Discussion

To ensure that the splice variants seen in murine melanopsin are not unique to this species [7], we sought confirmation for the existence of *Opn4* isoforms in other mammals. We found empirical evidence for *Opn4S* and *Opn4L* variants in humans (Figure S1), and bioinformatic analysis of genomic sequences indicates the presence of similar open reading frames in several other mammalian species including the chimpanzee *Pan troglodytes* and the opossum *Monodelphis domestica* (data not shown). Non-mammalian species have also been shown to possess multiple genes and splice variants of melanopsin, including chicken [8], *Xenopus*, and elephant shark [9]. Such findings argue that the splice variants of OPN4 in mice are unlikely to be unique to this species but are of functional significance across the vertebrates.

RNAi provides an acute and exquisitely specific tool to dissect the role of the different melanopsin isoforms in vivo [10]. We designed and tested siRNAs against each isoform of *Opn4* as well as a universal sequence that silenced both isoforms. We confirmed the successful delivery of siRNA to pRGCs, along with their efficacy and specificity both in vivo and in vitro (Figure S2). Following delivery, we studied the pupillary light response (PLR), negative masking, phase shifting of circadian rhythms of locomotor behavior, and light-induced sleep induction after silencing of both or either *Opn4* isoform in vivo.

**Figure 1:**
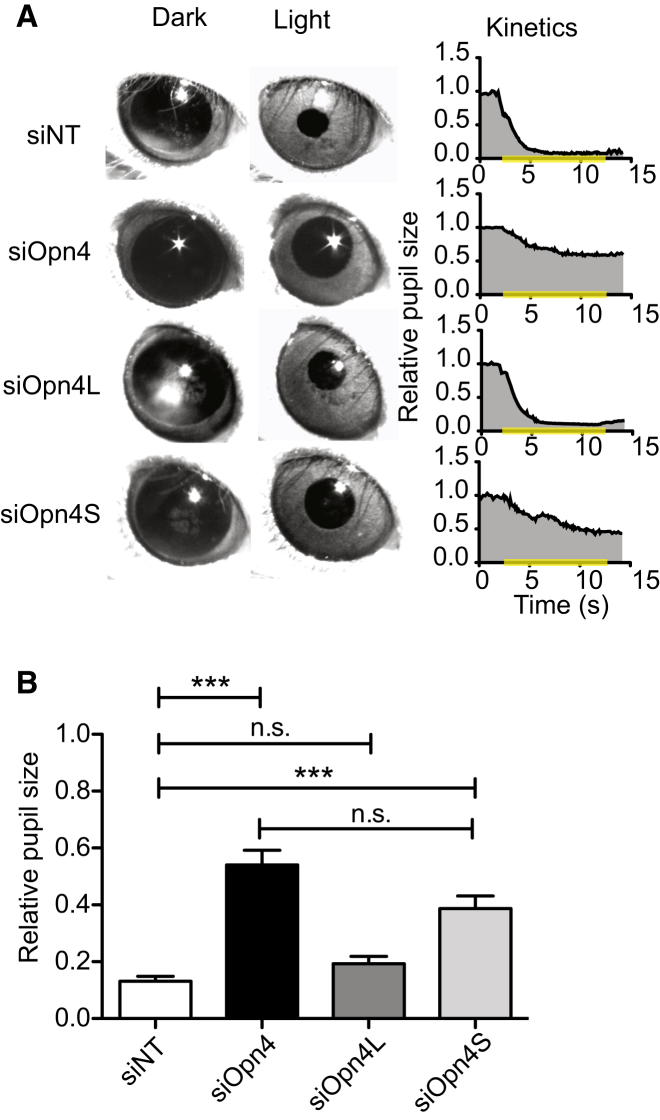
OPN4S Mediates the Pupillary Light Response Animals received siOpn4, siOpn4L, or siOpn4S in the one eye and siNT in the contralateral eye. The left eye was then stimulated by exposure to bright 480 nm light, and pupil constriction was imaged and measured from the right eye. (A) Images of pupil before (left panel) and immediately after (right panel) exposure to light for 10 s. Graph on right shows kinetics of pupil constriction, with pupil size normalized to dark level on the y axis. The yellow bar indicates duration of light exposure. Representative images and graphs for animals injected with siNT, siOpn4, siOpn4L, and siOpn4S are included. (B) Average pupil constriction at the end of the light pulse for animals injected with siRNA as indicated on the y axis, showing significantly attenuated pupil constriction for siOpn4 (0.58 ± 0.06 versus 0.13 ± 0.017; n = 7; p = 0.0001) and siOpn4S (0.39 ± 0.04; n = 10; p = 0.0002). siOpn4L did not significantly attenuate pupil constriction (0.19 ± 0.03; n = 10; p = 0.09), and siOpn4S treatment did not statistically differ from siOpn4 treatment (p = 0.08). ^∗^p < 0.05, ^∗∗^p < 0.01, ^∗∗∗^p < 0.001; one-way ANOVA with Tukey’s post-tests. Error bars represent the SEM. See also Figures S1, S2, and S4.

Melanopsin-deficient (*Opn4*^*−/−*^) mice show an attenuated PLR [5], and mice lacking rods, cones, and pRGCs (*Gnat1*^*−/−*^, *Cnga3*^*−/−*^, and *Opn4*^*−/−*^) show no PLR [2]. In *rd/rd cl* mice, which lack rods and cones, siRNA-mediated knockdown of *Opn4* should result in a substantially reduced PLR [10], as OPN4 is the only remaining photopigment [1]. We would not predict the complete loss of the PLR as siRNA knockdown is not complete in vivo (Figure S2) [11–14]. We found that knockdown of *Opn4* in the eye did indeed severely attenuate pupil constriction in response to light in the contralateral eye (Figure 1). Knockdown of *Opn4S* also resulted in a significant attenuation of the PLR, whereas knockdown of *Opn4L* had no effect (Figure 1), indicating that OPN4S provides the primary input of the light response driving the PLR.

**Figure 2:**
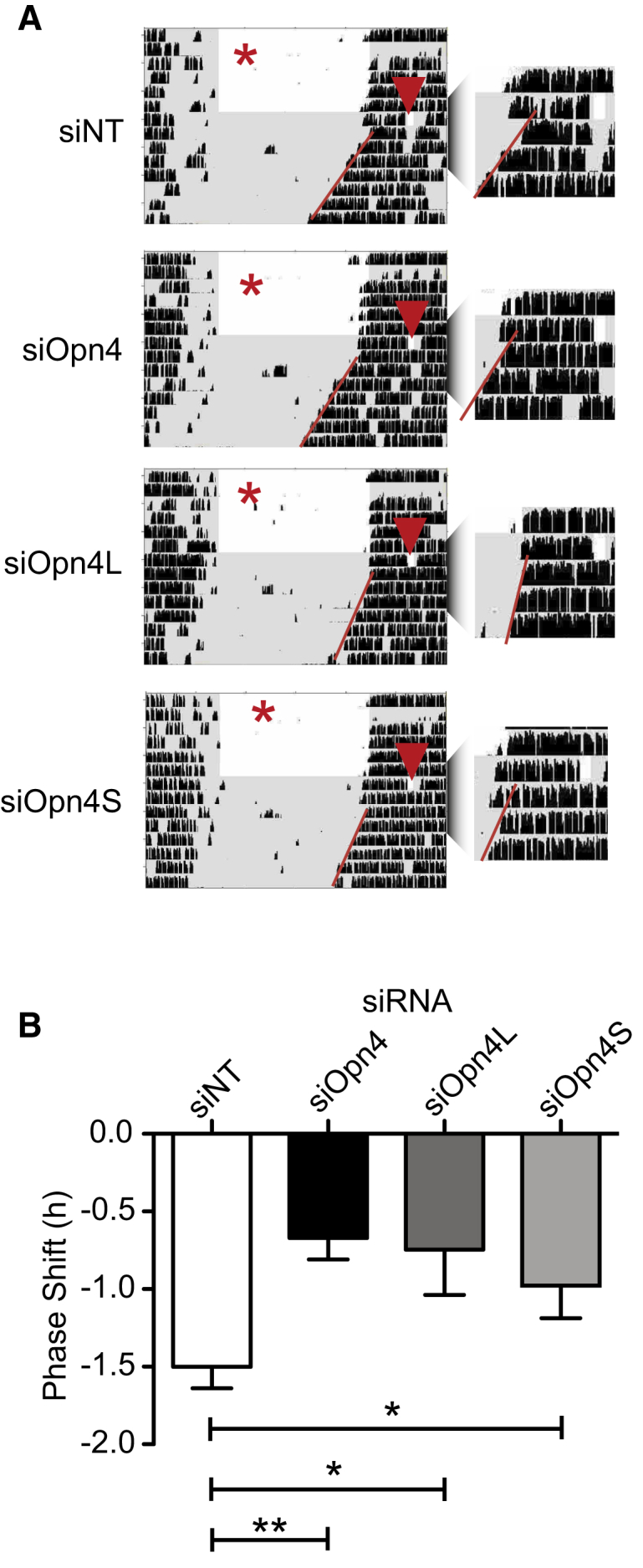
Both Isoforms of OPN4 Mediate Phase Shifting of Circadian Rhythms (A) Representative actograms from animals given intravitreal bilateral injection (indicated by a red star) of siNT, siOpn4, siOpn4S, and siOpn4L. Significantly reduced phase shifting after a 30 min CT16 light pulse (indicated by a red arrow) is seen for all three *Opn4*-targeting siRNAs versus siNT control. Actograms are enlarged around the light pulse for clarity. (B) Histogram showing average phase shift for siOpn4-treated animals (−0.68 ± 0.13 versus −1.50 ± 0.14 hr; n = 12; p = 0.0003) and siOpn4L and siOpn4S (−0.74 ± 0.29; p = 0.01; n = 8 and −0.98 ± 0.21; p = 0.04; n = 8, respectively). ^∗^p < 0.05, ^∗∗^p < 0.01; one-way ANOVA with Tukey’s post-tests. Error bars represent the SEM. See also Figures S1 and S2.

Phase shifting of circadian rhythms in response to a nocturnal light pulse has been shown previously to be attenuated in *Opn4*^−/−^ mice [3]. siRNA was administered bilaterally to achieve knockdown of either or both isoforms of *Opn4* in both eyes. Four days later, the animals received a 30 min light pulse at circadian time (CT) 16. *Opn4* knockdown resulted in smaller phase delays compared with the control injected with a non-targeting siRNA (reduced to 50%). Knockdown of either isoform caused a modest but significant reduction in the magnitude of phase shifts (Figure 2). These data indicate that light responses from both isoforms reach the SCN and mediate phase-shifting responses.

**Figure 3:**
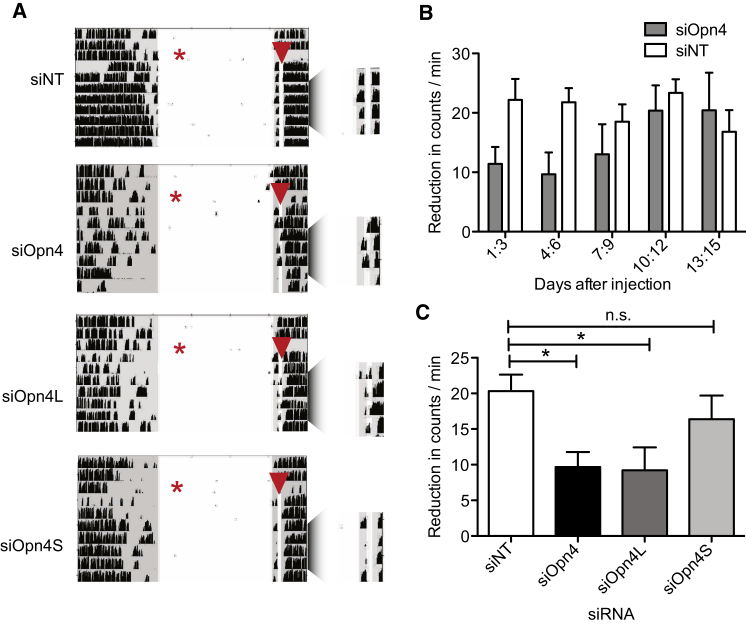
OPN4L Mediates Negative Masking (A) Representative actograms from animals given intravitreal bilateral injection (indicated by a red star) of siNT, siOpn4, siOpn4S, and siOpn4L show negative masking after a 10 min ZT12.5 light pulse (indicated by a red arrow) every day following injection. This protocol avoids significant light-induced phase shifts. Negative masking is attenuated with siOpn4 and siOpn4L. Actograms are enlarged around light pulse for clarity. (B) Histogram of reduction in activity during the light pulse (as compared with activity preceding the light pulse) across 3-day bins as indicated on the y axis. (C) Histogram of reduction in average (of days 2–8) wheel-running activity during the nocturnal light pulse shows smaller reductions in activity with siOpn4 (11.1 ± 1.6 versus 22.2 ± 2.3 siNT) and siOpn4L-treated animals (9.7 ± 3.6). siOpn4S had no significant effect (18.0 ± 3.4). n = 6. ^∗^p < 0.05; one-way ANOVA with Dunnett’s post-test. Error bars represent the SEM. See also Figures S1–S4.

We then proceeded to evaluate the effect of siRNA-mediated knockdown of *Opn4* on negative masking, another response that has been shown to be attenuated in *Opn4*^*−/−*^ mice [6]. After bilateral knockdown of *Opn4* or its isoforms, animals were given a 10 min light pulse at half an hour after lights off (ZT 12.5) every day, which was designed to avoid the phase-shifting effects of light. Knockdown of both isoforms resulted in attenuated negative masking during the 10 min light pulse, similar to that seen in *Opn4*^*−/−*^ mice [6] (Figures 3A and 3B). Knockdown of *Opn4S* had no significant effect on negative masking, whereas *Opn4L* silencing attenuated this response, to the same degree as knockdown of *Opn4* itself (siOpn4L = 75 ± 14; siOpn4 = 62 ± 8; p = 0.36; Figures 3A, 3C, and S3). These results led us to conclude that light signaling by the long isoform of OPN4 is responsible for negative masking.

**Figure 4:**
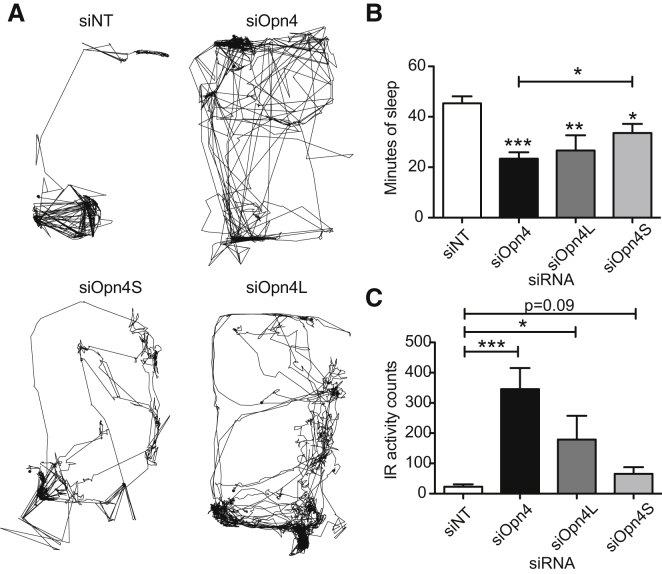
Both Isoforms of OPN4 Mediate Sleep Induction (A) Animals were given bilateral siRNA (siNT, siOpn4, siOpn4S, and siOpn4L) injections and, 4 days later, given a 1 hr light pulse at ZT14, during which videos were recorded and analyzed. The traces show activity patterns during the light pulse of individually housed mice receiving siRNAs as indicated. siNT-injected animals restricted their movements to their nest, where they spent the majority of time sleeping. The *Opn4* knockdown animals showed markedly decreased levels of sleep. (B) Total sleep levels (measured as bouts of >40 s immobility) were measured [21] during the course of the light pulse. *Opn4* knockdown animals show severely attenuated sleep induction during the light pulse (23.38 ± 2.5 min versus 45.34 ± 2.8 min; n = 10; p < 0.0001). *Opn4L* and *Opn4S* knockdown animals also showed reduced levels of sleep (26.65 ± 6.0; n = 10; p < 0.01 and 33.58 ± 3.6; n = 10; p = 0.02). (C) Locomotor activity during the light pulse as measured via passive infrared recordings (PIR) for the same animals as above showing attenuated reductions in the case of *Opn4* and *Opn4L* knockdown (95.4% ± 30.7% and 35.4% ± 11.9% versus 7.4% ± 4.1% for siNT, respectively). Locomotor activity as measured by PIR during the first 10 min of the light pulse to compare with Figure 3 are provided in Figure S3C, and activity during the hour preceding the light pulse shows no significant differences across the groups as indicated in Figure S3D. ^∗^p < 0.05, ^∗∗^p < 0.01, ^∗∗∗^p < 0.001; one-way ANOVA with Tukey’s post-tests. Error bars represent the SEM. See also Figures S1–S3.

Our studies [4], and those of others [15–17], have shown OPN4-mediated signaling plays an important role in the regulation of sleep-wake states. pRGCs can regulate this response via a direct but sparse innervation to the ventrolateral preoptic nuclei [18, 19] (VLPO; the sleep switch) or via a substantial relay system arising from the SCN consisting of the vSPZ (sub-para-ventricular zone) and the DMH (dorsomedial hypothalamus) [20]. In view of the findings presented in Figure 2 that both OPN4L and OPN4S signal light to the SCN and that light via the SCN plays an important role in sleep regulation, we predicted that both splice variants would be important in regulating light-induced sleep. This prediction proved correct. Bilateral knockdown for *Opn4* and its isoforms was undertaken. Four days later, mice were exposed to a 1 hr light pulse at ZT 14, during which sleep was measured using video monitoring [21]. Simultaneous passive infrared recordings were made to assess activity levels. Knockdown of both isoforms results in attenuated induction of sleep, with knockdown animals showing higher locomotor activity (Figure 4A) and a 50% reduction in sleep during the 1 hr light pulse compared with the control (Figure 4B). Knockdown of either *Opn4S* or *Opn4L* also reduced the levels of sleep (Figure 4B). Activity in the *Opn4* knockdown animals was much higher than the control, as expected, and this was also seen after *Opn4L* knockdown (Figures 4A, 4C, and S3), consistent with the negative masking results described in Figure 3. Animals with knockdown of *Opn4S* show attenuated activity during the light pulse (Figures 4A and 4C), although the animals showed reduced sleep, showing that strong light-aversive responses [22] remained in these animals whereas the propensity to sleep was attenuated.

Recent work has demonstrated a role for PLCB4 (1-phosphatidylinositol-4,5-bisphosphate phosphodiesterase beta-4) in the melanopsin-signaling cascade [23], presumably acting downstream of Gnaq/11 type G proteins [24]. To determine whether both isoforms signal via the same cascade, we undertook *Plcb4* silencing (Figure S4A). Silencing attenuated the PLR (Figures S4B and S4C) and also negative masking (Figures S4D and S4E), mirroring the effect of *Opn4* silencing, indicating that PLCB4 participates in the signaling cascade of both OPN4S and OPN4L isoforms.

Here, we show that OPN4 isoforms mediate different behavioral responses to light, such that OPN4S mediates the PLR; OPN4L negative masking and both isoforms mediate phase shifting of locomotor behavior and sleep induction in response to light. Further, both isoforms signal via PLCB4. OPN4L and OPN4S differ only at their C-terminal tails, and thus, it seems likely that any functional differences between these isoforms must reside within these regions [7]. Bioinformatic analysis indicates that the longer tail of OPN4L may contain additional phosphorylation sites, and these may confer functional differences in responses mediated by OPN4L and OPN4S, most likely influencing rates of adaptation, recovery, and sensitization, as has been shown for other G-protein-coupled receptors, where phosphorylation of the C-terminal tail can significantly change receptor signaling [25, 26].

To date, at least five distinct subtypes of pRGC have been described, termed M1–M5 [27, 28]. These cell types project to different regions of the brain [28] and exhibit light responses with markedly different kinetics [29]. However, whereas it may be logical to conclude that different pRGC subtypes mediate different non-image-forming (NIF) responses to light on the basis of their anatomical projections [27], empirical evidence is largely lacking. Indeed, the only study to show a direct link between pRGC subtype and behavior is from Chen et al. [30]. Specifically, they showed that a subpopulation of *Brn3b*-negative M1 pRGCs that project to the SCN are capable of driving circadian entrainment following the ablation of all other pRGCs (*Brn3b*-positive and including M1–M5 pRGCs) [30]. Ablation of *Brn3b*-positive M1–M5 pRGCs was shown to disrupt the PLR, yet given the widespread loss of pRGC subtypes using this approach, and the complete loss of OPN innervations, it is not possible to conclude from this study which class of pRGC mediates the PLR or other NIF responses to light.

An intuitive explanation for the differences we describe would be the differential expression of OPN4L and OPN4S isoforms in different pRGC subtypes. However, whereas this may provide a partial explanation for our findings, it cannot provide the complete answer. For example, here, we show that the PLR is mediated by OPN4S, which is only expressed in M1 pRGCs [7]. M1 cells co-express OPN4L and OPN4S [7], and we show that silencing *Opn4L* produces no significant change in PLR (Figure 1). Thus, for pupil constriction, OPN4L cannot compensate for the loss of OPN4S in M1 pRGCs. We have shown that Opn4L is expressed at much-lower levels in the retina (about 40-fold less than *Opn4S*) [7]. Whereas silencing *Opn4L* would specifically target M2 cells (which express just *Opn4L*), it may have little effect on M1 cells, which will still express high levels of *Opn4S*. It is entirely plausible that this may be sufficient to drive a pupillary response. In addition, it is also possible that the two isoforms dimerize/oligomerize in different combinations, and these hetero/homo oligomers have different signaling properties. This has been demonstrated amply with several other GPCRs (see Palczewski et al., 2010 [31] for review on rhodopsin oligomerization). Hetero oligomerization of GPCRs can result in differences in pharmacology and downstream signaling pathways and also modulate the strength of the signal. For example, heterodimerization of the opioid δ/κ receptors results in signaling potentiation [32] and heterodimerization is required for transactivation of the GABA receptors GB1 and GB2 [33].

Collectively, our findings demonstrate that splice variants of the melanopsin gene can regulate strikingly different behaviors. In addition to highlighting the diversity of melanopsin signaling, these data provide one of the very few examples we have across the animal kingdom that isoforms of a single gene can regulate highly divergent behaviors. Furthermore, this is the only example in visual biology that naturally occurring opsin isoforms mediate different physiological and behavioral responses to light.

## Experimental Procedures

All animals used were retinal degenerate *rd/rd* (C3H/HeN; Harlan UK) mice (older than 80 days) lacking rod and the majority of cone photoreceptors, unless otherwise indicated as *rd/rd cl* [1]. All animals were housed under a 12:12 LD cycle with food and water ad libitum. All procedures were conducted in accordance with the Animals (Scientific Procedures) Act 1986 and the University of Oxford Policy on the Use of Animals in Scientific Research (PPL 70/6382 and 30/2812). All procedures were reviewed by the Clinical Medicine Animal Welfare and Ethical Review Body (AWERB). Animals were sacrificed via schedule 1 methods in accordance with the UK Home Office Animals (Scientific Procedures) Act 1986.

## Author Contributions

A.J. performed all in vivo RNAi experiments and wrote the manuscript with input from S. Hughes, R.G.F., and S.N.P. S. Hughes performed the immunohistochemistry and initial in vitro RNAi experiments. A.A. designed the isoform-specific siRNAs with input from M.J.A.W. M.H. designed and provided siRNAs suitable for in vivo use. C.A.P., S.S.P., V.P., L.A.B., A.V., and R.E.M. assisted with in vivo experiments. S.S.P. and S. Halford performed the studies with human OPN4 isoforms. S.G., M.W.H., M.J.A.W., R.G.F., and S.N.P. provided input into the conception of the project and all aspects of experimental work. S.N.P. and R.G.F. reviewed and edited the manuscript.

## Accession Numbers

The accession number for the human Opn4L sequence reported in this paper is GenBank: KT381095.

## Supplemental Information

Document S1. Supplemental Experimental Procedures and Figures S1–S4

Document S2. Article plus Supplemental Information
