# Supplementary material for: Isoforms of Melanopsin Mediate Different Behavioral Responses to Light
Source: Curr Biol. 2015 Sep 21;25(18):2430–4. doi: 10.1016/j.cub.2015.07.071 (PMC4580334; doi:10.1016/j.cub.2015.07.071)
Supplement: Document S1. Supplemental Experimental Procedures and Figures S1–S4 [file mmc1.pdf]

Current Biology
Supplemental Information

Isoforms of Melanopsin Mediate Different Behavioral Responses to Light

Aarti Jagannath, Steven Hughes, Amr Abdelgany, Carina A. Pothecary, Simona Di Pretoro, Susana S. Pires, Athanasios Vachtsevanos, Violetta Pilorz, Laurence A. Brown, Markus Hossbach, Robert E. MacLaren, Stephanie Halford, Silvia Gatti, Mark W. Hankins, Matthew J.A. Wood, Russell G. Foster, and Stuart N. Peirson

Figure S1

A

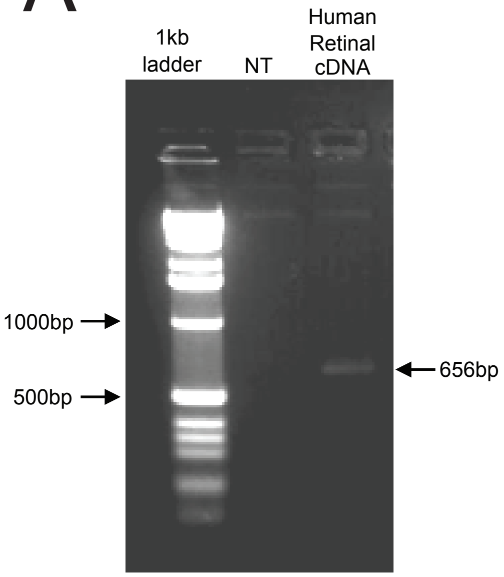

B

```
       HOPN47F                         Exon7
GCCAAGGCCTCTGCAATCCACAACCCCATCATTTACGCCATCACCCACCCCAAGTACAGG   60
A  K  A  S  A  I  H  N  P  I  I  Y  A  I  T  H  P  K  Y  R
GTGGCCATTGCCCAGCACCTGCCCTGCCTGGGGGTGCTGCTGGGTGTATCACGCCGGCAC  120
V  A  I  A  Q  H  L  P  C  L  G  V  L  L  G  V  S  R  R  H
                                      Exon8
AGTCGCCCCTACCCCAGCTACCGCTCCACCCACCGCTCCACGCTGACCAGCCACACCTCC  180
S  R  P  Y  P  S  Y  R  S  T  H  R  S  T  L  T  S  H  T  S
AACCTCAGCTGGATCTCCATACGGAGGCGCCAGGAGTCCCTGGGCTCGGAGAGTGAGGTG  240
N  L  S  W  I  S  I  R  R  R  Q  E  S  L  G  S  E  S  E  V
                                      Exon9
GGCTGGACACACATGGAGGCAGCAGCTGTGTGGGGAGCTGCCCAGCAAGCAAATGGGCGG  300
G  W  T  H  M  E  A  A  A  V  W  G  A  A  Q  Q  A  N  G  R
TCCCTCTACGGTCAGGGTCTGGAGGACTTGGAAGCCAAGGCACCCCCCAGACCCCAGGGA  360
S  L  Y  G  Q  G  L  E  D  L  E  A  K  A  P  P  R  P  Q  G
CACGAAGCAGAGACTCCAGGGAAGGTGACTGGGCCCGGTACCTGCCAATCCACAAAGGGG  420
H  E  A  E  T  P  G  K  V  T  G  P  G  T  C  Q  S  T  K  G
TGGGGGTTAGGGGCCAGTAGCCCAGGGAGAGGCCAGGAAAGAGAGACTTGTTCTCATGGG  480
W  G  L  G  A  S  S  P  G  R  G  Q  E  R  E  T  C  S  H  G
CAGGGGCGATATCCTCCAGGAATCACCTGCTCCCAGCACCTCCCAGCTTTTCCTTGTCTG  540
Q  G  R  Y  P  P  G  I  T  C  S  Q  H  L  P  A  F  P  C  L
CCTGGGGTTCTCTGTGACTCTGAGATGCTCTGAgcagggcctgcatatggtctgcacatg  600
P  G  V  L  C  D  S  E  M  L  *
tgtgtgtgtgtgtgtgatcatgcaactctgactctgcatggtgctgttggctgtgc        656
                                             HOPN4Long
```

C

```
HumanLong     AKASAIHNPIIYAITHPKYRVAIAQHLPCLGVLLGVSRRHSRPYPSYRSTHRSTLTSHTS 60
MouseLong     AKASAIHNPIIYAITHPKYRVAIAQHLPCLGVLLGVSGQRSHPSLSYRSTHRSTLSSQSS 60
HumanShort    AKASAIHNPIIYAITHPKYRVAIAQHLPCLGVLLGVSRRHSRPYPSYRSTHRSTLTSHTS 60
MouseShort    AKASAIHNPIIYAITHPKYRVAIAQHLPCLGVLLGVSGQRSHPSLSYRSTHRSTLSSQSS 60
              ************************************* ::*:*  **********:*::*!

HumanLong     NLSWISIRRRQESLGSESEVGWTHMEAAAVWGAAQQANGRSLYGQGLEDLEAKAPPRPQG 120
MouseLong     DLSWISGRKRQESLGSESEVGWTDTETTAAWGAAQQASGQSFCSQNLEDGELKASSSPQV 120
HumanShort    NLSWISIRRRQESLGSESEVGWTHMEAAAVWGAAQQANGRSLYGQGLEDLEAKAPPRPQG 120
MouseShort    DLSWISGRKRQESLGSESEVGWTDTETTAAWGAAQQASGQSFCSQNLEDGELKASSSPQV 120
              :***** *:**************. *::*.*******.*:*: .*.*** * **.. ** 

HumanLong     HEAETPGKVTGPGTCQSTKGWGLGASSPGRGQE-RETCSHGQGRYPPGITCSQHLPAFPC 179
MouseLong     QRSKTP-KVPGPSTCRPMKGQGARPSSLRGDQKGRLAVCTGLSECPHPHT-SQFPLAF-- 176
              :.::** **.**.**:. ** *  .**   .*: * : . * .. *   * **.  ** 
HumanShort    HEAETPGKTKGLIPSQDPRM                                         140
MouseShort    QRSKTP-KTKGHLPSLDLGM                                         139
              :.::** **** :** *  *

HumanLong     LPGVLCDSEML                                                  190
MouseLong     LEDDVTLRH-L                                                  186
              * . :   . *
```

Figure S2

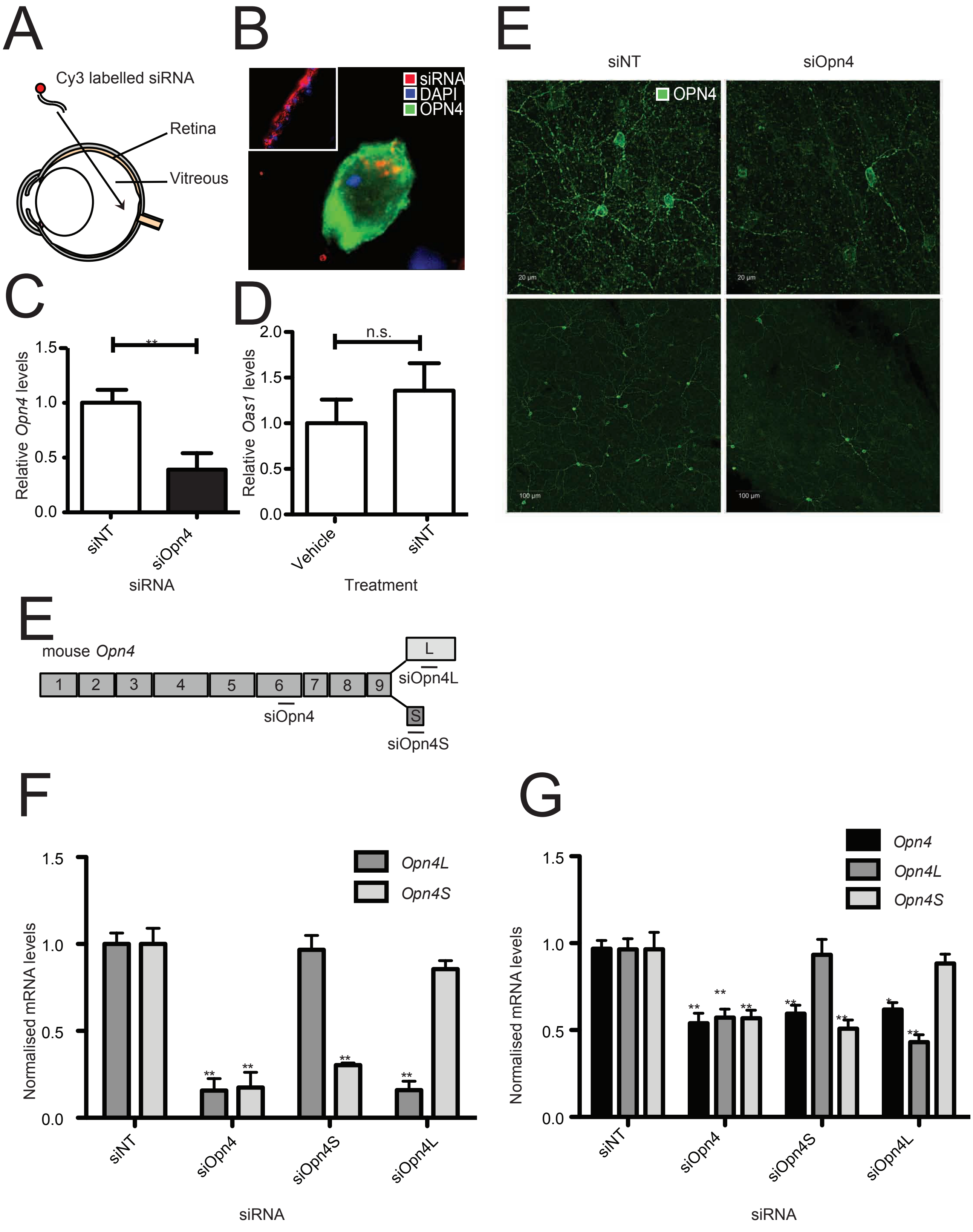

A
Cy3 labelled siRNA
Retina
Vitreous
B
siRNA
DAPI
OPN4
E
siNT
siOpn4
OPN4
C
Relative Opn4 levels
**
siNT
siOpn4
siRNA
D
Relative Oas1 levels
n.s.
Vehicle
siNT
Treatment
E
mouse Opn4
1 2 3 4 5 6 7 8 9
L
S
siOpn4L
siOpn4
siOpn4S
F
Normalised mRNA levels
Opn4L
Opn4S
siNT
siOpn4
siOpn4S
siOpn4L
siRNA
G
Normalised mRNA levels
Opn4
Opn4L
Opn4S
siNT
siOpn4
siOpn4S
siOpn4L
siRNA

Figure S3

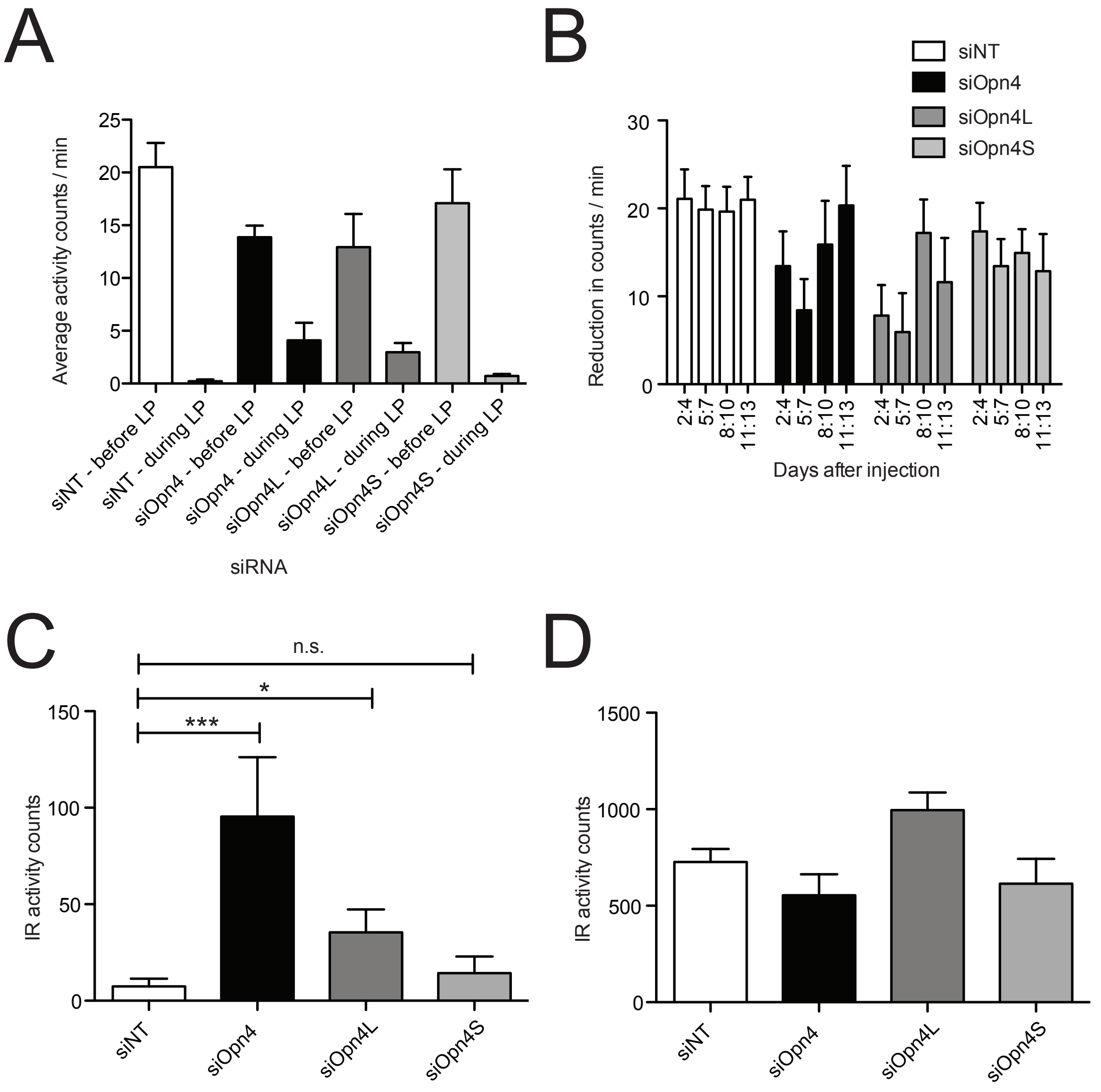
A
B
siNT
siOpn4
siOpn4L
siOpn4S
Average activity counts / min
25
20
15
10
5
0
siNT - before LP
siNT - during LP
siOpn4 - before LP
siOpn4 - during LP
siOpn4L - before LP
siOpn4L - during LP
siOpn4S - before LP
siOpn4S - during LP
siRNA
Reduction in counts / min
30
20
10
0
2:4
5:7
8:10
11:13
Days after injection
C
D
n.s.
*
***
IR activity counts
150
100
50
0
siNT
siOpn4
siOpn4L
siOpn4S
IR activity counts
1500
1000
500
0
siNT
siOpn4
siOpn4L
siOpn4S

Figure S4

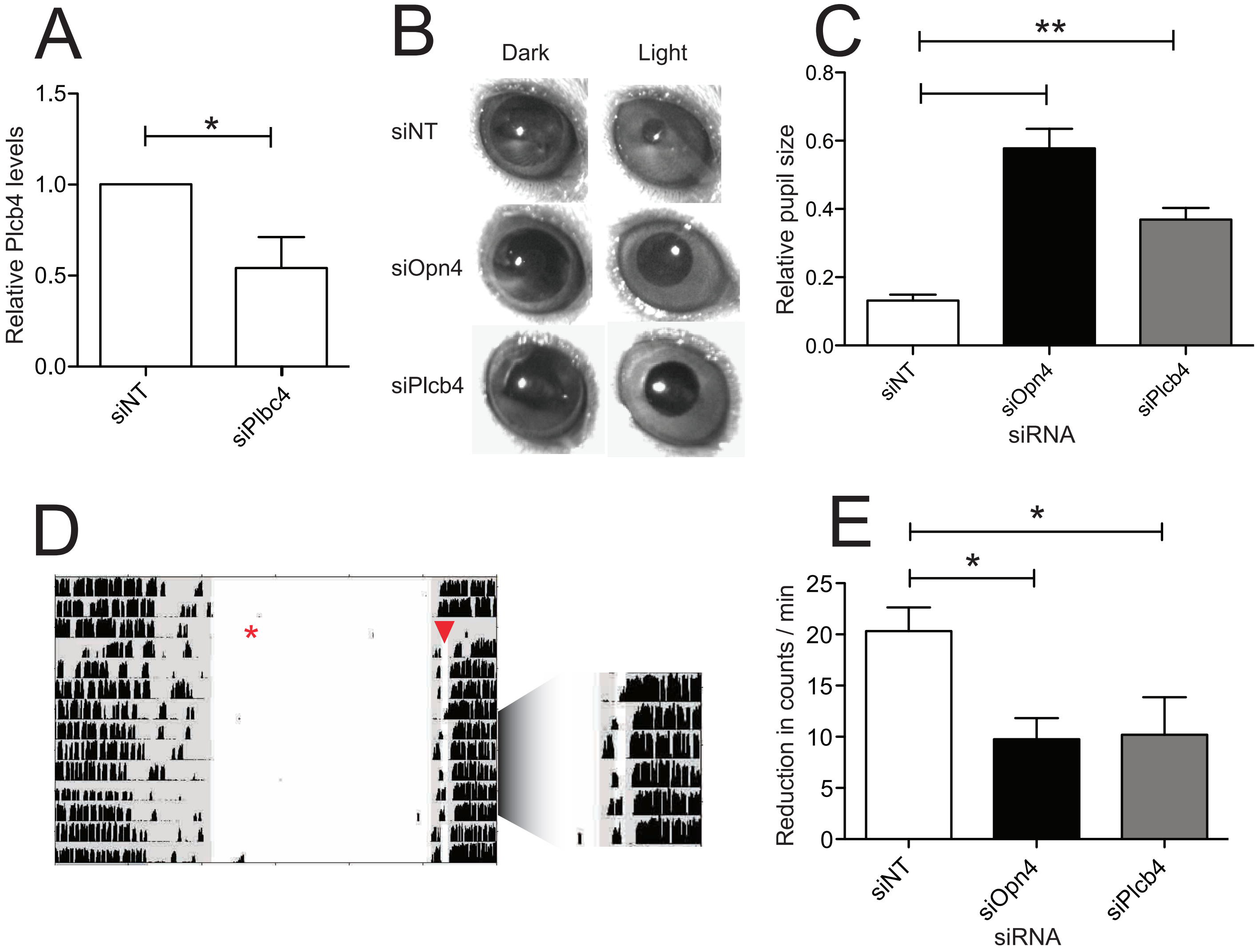

A
B
C
D
E
Relative Plcb4 levels
1.5
1.0
0.5
0.0
*
siNT
siPlbc4
Dark
Light
siNT
siOpn4
siPlcb4
**
0.8
0.6
0.4
0.2
0.0
Relative pupil size
siNT
siOpn4
siPlcb4
siRNA
*
*
Reduction in counts / min
25
20
15
10
5
0
siNT
siOpn4
siPlcb4
siRNA

Supplemental Figure Legends

Figure S1: Evidence for human *Opn4L* relating to Figs. 1-4.

A) Amplification of a partial fragment of human OPN4 long from retinal cDNA using primers HOPN47F and HOPN4Long. 1kb Ladder (Invitrogen). **B)** Nucleotide sequence and deduced amino acid sequence of 656bp PCR product generated using primers HOPN47F and HOPN4Long (sequences are shaded) from human retinal cDNA. The intron- exon boundaries are marked with vertical blue lines. The product spans exons 7, 8 and 9 and reads through the predicted end of exon 9. **C)** Clustal W alignment of the predicted amino acid sequences of human and mouse long and short isoforms. Sequences are shown from the start of primer HOPN47F. The long isoforms show 70% similarity over this sequence and the short isoforms 84% similarity.

Figure S2: siRNA-mediated silencing of *Opn4* relating to Figs. 1-4.

A) Schematic showing intravitreal injection of chemically stabilized Cy3-labelled siRNA. **B)** siRNA successfully reached the ganglion cell layer (inset), and was identified within melanopsin-expressing photosensitive retinal ganglion cells, OPN4 immunostained in green, siRNA in red and nuclei with DAPI in blue. **C)** qPCR for *Opn4* mRNA showing significant silencing in the animals treated with siRNA against *Opn4* (siOpn4) vs non-targeting siRNA (siNT) (55%, p=0.006, n=10). **D)** qPCR for *Oas1* shows no significant induction of this gene in siRNA treated retina (siNT) vs vehicle treated retina (vehicle), showing the absence of an interferon response; one of the prime concerns *in vivo* RNAi [29,30], n=6 for each group, *=p<0.05, **=p<0.01 **E)** Whole retinal flat-mounts showing markedly lower levels of OPN4 (green) in siOPN4 transfected retina. **F)** Schematic of the *Opn4* gene, showing the target position of the siRNAs against both *Opn4* isoforms (siOpn4), *Opn4L* (siOpn4L) and *Opn4S* (siOpn4S). siOpn4 matches an area in exon6, siOpn4L a region of exon 9 specific to *Opn4L* and siOpn4S exon 10, specific to *Opn4S*. **G)** qPCR of *Opn4L* and *Opn4S* from stably transfected Neuro2A cell lines expressing each isoform, treated with siRNA as indicated, showing specific silencing of each isoform by the respective siRNA. N=6, **=p<0.01, T-test. **H)** Similar results are seen *in vivo; Opn4* levels are measured with 3 primers, the

first targeting a fragment in the 5’ region, thereby covering both isoforms (*Opn4*, white), the second targeting *Opn4S* (*Opn4S*, light grey), the third targeting *Opn4L* (*Opn4L*, dark grey). N=16, **=p<0.01, *=p<0.05, ANOVA with post-hoc Dunnett’s tests.

Figure S3: Wheel running activity during light pulse relating to Fig. 3 and activity measurements to Fig. 4

A) Histogram of total activity before and during the 10 minute light pulse averaged through days 2-8 after injection for the four different siRNA treatments. **B)** reduction in activity during the light pulse from an equivalent period just before the pulse across 3 day bins for the four siRNAs as above.

C) Locomotor activity as measured by PIR during the first 10 minutes of the light pulse, to compare with Fig. 3 *Opn4* and *Opn4L* knockdown significantly reduce masking. **D)** Activity as measured by PIR during the hour preceding the light pulse shows no significant differences (n=4, p=0.1, one way ANOVA) across the groups, showing sleep pressure is not a contributing factor to levels of sleep induction.

Figure S4: PLCB4 participates in OPN4L and OPN4S signalling cascades relating to Figs. 1 and 3.

A) Silencing of *Plcb4* mRNA *in vivo* (54% of control, p=0.049, n=5). **B)** Pupil images before and immediately after a 10s exposure to bright 480nm light, showing reduced pupil constriction with siOpn4 and siPlcb4. **C)** Histogram of average pupil constriction at the end of the light pulse for animals injected with siRNA as indicated, showing significantly attenuated pupil constriction for siPlcb4 (0.37 ± 0.03 n=12, p<0.0001 vs siNT). siOpn4 values provided for comparison **D)** Representative actogram from an animal given intravitreal bilateral injection (indicated by a red star) of siPlcb4 showing low levels of negative masking during a 10 min ZT12.5 light pulse (indicated by a red arrow) every day following injection. Actograms enlarged around light pulse for clarity. **E)** Histogram of reduction in wheel running activity during the nocturnal 10 min light pulse shows severely attenuated masking with siPlcb4 (83.53±12.76 n=6 vs 30.42±5.89 n=10 siNT, p=0.001) and siOpn4 values provided for comparison. *=p<0.05, **=p<0.01

Supplemental Experimental Procedures

Animals: All animals used were retinal degenerate *rd/rd* (C3H/HeN, Harlan UK) mice (older than 80 days) lacking rod and the majority of cone photoreceptors, unless otherwise indicated as *rd/rd cl* [S1]. All animals were housed under a 12:12 LD cycle with food and water ad libitum. All procedures were conducted in accordance with the Animals (Scientific Procedures) Act 1986 and the University of Oxford Policy on the Use of Animals in Scientific Research (PPL 70/6382 and 30/2812). All procedures were reviewed by the Clinical Medicine Animal Welfare and Ethical Review Body (AWERB). Animals were sacrificed via Schedule 1 methods in accordance with the UK Home Office Animals (Scientific Procedures) Act 1986.

Behavioural tests:

Pupillometry: Assessment of pupillary light responses was performed 72 hours post intravitreal injection of siRNA, using methods reported previously [S2]. Testing was performed between ZT 4-8, and all animals were dark adapted for 1–2 hours prior to testing. A xenon arc lamp (150W solar simulator, Lot Oriel, UK) with a 480 nm monochromatic filter (Andover, 10 nm half-bandwidth) was used to produce a light intensity of 14.6 log quanta/cm^2/s (173 μW/cm^2/s). Light stimuli (10 seconds) were transmitted to the eye via a liquid light pipe as an irradiant light stimulus using a 2” integrating sphere (Pro-lite Technology, UK) and was controlled by a shutter positioned in the light path (LSZ160 shutter, Lot Oriel UK; custom software supplied by BRSL, Newbury, UK). Images of consensual pupil responses were collected with a Prosilica NIR sensitive CCD video camera (BRSL, Newbury, UK) at a rate of 10 frames per second, under infrared LED illumination (850nm, 10nm half-bandwidth). During pupil measurements unanaesthetised animals were temporarily restrained using normal husbandry techniques for the duration of the recording (29 seconds, including baseline, stimulation and recovery phases). Each animal was tested 5 times to minimise any artefacts due to handling. Data reported for each individual animal represents the mean of all

individual trials. All images were analysed using ImageJ software (NIH; rsbweb.nih.gov/ij/).

Phase Shifting: Mice were maintained on running wheels in light tight chambers on a 12:12 LD cycle (100 lux from white LED lamps) and received a bilateral injection of the indicated siRNA. 3 days after the injection, the mice were placed in DD for 24 hours and received a 30 min light pulse at approximately CT16. The mice were then allowed to free run for 14 days or longer in DD, running wheel activity data were collected and analysed on Clocklab (Actimetrics, Wilmette, IL).

Masking: Mice were housed under a 12:12 LD cycle (400 lux from SpectraNova halogen white light (Schott AG, Mainz, Germany) in light tight chambers and received a bilateral injection of the indicated siRNA. The day following the injection onwards, the mice also received a 10 min light pulse at ZT12.5 every day for the following 20 days. Activity data were collected and analysed on Clocklab.

Sleep: Mice were housed under a 12:12 LD cycle (400 lux from SpectraNova halogen white light (Schott AG, Mainz, Germany) in light tight chambers and received a bilateral injection of the indicated siRNA. 4 days later, the mice were given a 1 hour light pulse at ZT14 during which videos of each mouse were recorded. Sleep was then analysed from the videos as bouts of immobility >40s [S3]. Simultaneous activity recordings were made using passive infrared monitors. Activity patterns within the cage were analysed using Anymaze (www.anymaze.com).

***In vitro* RNAi and siRNA sequences:** We used chemically stabilised siRNAs previously shown to be suitable for in vivo use [S4, S5]. siRNA sequences against *Opn4* were purchased predesigned from Invitrogen as Stealth siRNAs. siOpn4L and siOpn4S were designed by hand and ordered from Dharmacon. Silencing efficiency was tested in vitro using Neuro2A cells stably expressing either *Opn4S* or *Opn4L* as previously described [S6]. siRNAs were transfected at the indicated levels (between 5 and 50nM) using Lipofectamine RNAi max according to manufacturer's instructions. Knockdown was assessed after 48 hours. Non-targeting siRNA (siNT) was purchased from Dharmacon.

siOpn4: 5’ CACUGAUUGUCAUUCUUCUUU 3’
siOpn4L: 5’ GUAGCCUAAGGGGUGACCAUU 3’
siOpn4S: 5’ AGUCUGGAUCUCGGGAUGUAGUU 3’
siNT: 5’ CUUACGCUGAGUACUUCGAUU 3’
siPLCB4: 5’ GGAAGUAAUUGUCUUGAAAUU 3’

***In vivo* RNAi:** siRNA sequences validated in vitro were purchased as HPLC purified backbone modified sequences designed to be stabilized for in vivo use (siSTABLE, Dharmacon). siRNAs were complexed with Invivofectamine (Invitrogen2.0) for *in vivo* delivery according to manufacturer’s instructions. Animals were aneasthetized with ketamine:meditomidine. 3 ug of siRNA was delivered into each eye in a final volume of 1.5 ul, injected intravitreally with a Hamilton syringe (Hamilton Company) with a 34 gauge 10mm point style 2 needle.

mRNA extraction, qPCR and real-time PCR primer sequences: Total RNA was exracted from Neuro2A cells using the RNEasy mini kit (Qiagen) and from retina using the RNEasy Mini kit (following instructions for mRNA extraction), cDNA was prepared using the qScript cDNA synthesis kit (Quanta Biosciences) according to manufacturer’s instructions. Real-time PCR was performed as described previously [S7] using a SYBR green master mix (Qiagen Fast SYBR) with gene specific primers described previously [S7, S8].

Amplification of human *OPN4* long isoform

To confirm if a similar splicing mechanism is also seen in human the 3’ region of the human melanopsin gene (Genbank accession number AF147788) was analysed and a forward primer was designed in Exon 7 (H*OPN4*7F 5’-GCCAAGGCCTCTGCAATCCA-3’) and a reverse primer designed to the 3’ untranslated region of the predicted long isoform (H*OPN4*Long 5’-CATGGTGCTGTTGGCTGTGC-3’). PCR was performed using Platinum *Taq* Supermix (Invitrogen) with an initial denaturation step at 94°C for 3 min, then 94°C for 30 s, 57°C for 30 s, and 72°C for 1 min 30 s for 35 cycles, followed by a final extension at 72°C for 7 min. PCR was carried out using human retinal cDNA (Clontech) as template with Platinum PCR Supermix (Invitrogen)

and each 25µl reaction contained 0.2µM each primer and 0.5µl of cDNA (0.5ng). The 656bp fragment obtained was cloned into pGEM-T Easy and sequenced.

Immunohistochemistry:

Immunostaining of retina cryostat sections and whole retina flatmounts was performed as described previously [S2, S9]. Briefly, retinal sections were permeabilised in PBS with 0.2% Triton X at RT for 20 min and blocked in PBS with 10% normal donkey serum (Sigma) with 0.2% Triton X for 1 hour at RT. Rabbit polyclonal anti-melanopsin antibody recognising the N-terminus of murine Opn4common to both Opn4L and Opn4S (UF006, Advanced Targeting Systems) was diluted 1:2500 and incubated for 16 hours at 4°C diluted in 2.5% donkey serum in PBS with 0.2% Triton X. Donkey anti-rabbit Alexa-488 and Alexa-568 secondary antibodies (Life Technologies) were incubated for 2 hours at RT diluted 1:200 in 2.5% donkey serum in PBS with 0.2% Triton X. All wash steps were performed using PBS with 0.05% Tween-20. Sections were mounted in Prolong Gold anti-fade reagent containing DAPI (Life Technologies). Similar methods were employed for whole retina staining, although primary antibody was incubated for 3 days and levels of Triton X were increased to 1% for all steps. Fluorescent images were acquired using an inverted LSM 710 laser scanning confocal microscope (Zeiss) with Zen 2010 image acquisition software (Zeiss). Excitation was 405nm, 488nm and 561nm with emissions collected between 440-480, 505-550 and 600-700nm for DAPI, green and red fluorescence respectively. For all images, global enhancements of brightness and contrast were performed using ImageJ software. For comparative analysis all images were collected and processed under identical conditions.

Statistical tests:

All data were analysed and plotted on GraphPad Prism. Statistical comparisons were made using one-way ANOVA followed by Tukey's multiple comparison tests.

References:

S1. M. S. Freedman, R. J. Lucas, B. Soni, M. von Schantz, M. Munoz, Z. David-Gray, R. Foster, Regulation of mammalian circadian behavior by non-rod, non-cone, ocular photoreceptors. *Science* **284**, 502-504 (1999)

S2. S. Hughes, C. A. Pothecary, A. Jagannath, R. G. Foster, M. W. Hankins, S. N. Peirson, Profound defects in pupillary responses to light in TRPM-channel null mice: a role for TRPM channels in non-image-forming photoreception. *Eur J Neurosci* **35**, 34-43 (2012)

S3. S. P. Fisher, S. I. Godinho, C. A. Pothecary, M. W. Hankins, R. G. Foster, S. N. Peirson, Rapid assessment of sleep-wake behavior in mice. *J Biol Rhythms* **27**, 48-58 (2012)

S4. A. L. Jackson, J. Burchard, D. Leake, A. Reynolds, J. Schelter, J. Guo, J. M. Johnson, L. Lim, J. Karpilow, K. Nichols, W. Marshall, A. Khvorova, P. S. Linsley, Position-specific chemical modification of siRNAs reduces "off-target" transcript silencing. *RNA* **12**, 1197-1205 (2006)

S5. F. Czauderna, M. Fechtner, S. Dames, H. Aygun, A. Klippel, G. J. Pronk, K. Giese, J. Kaufmann, Structural variations and stabilising modifications of synthetic siRNAs in mammalian cells. *Nucleic Acids Res* **31**, 2705-2716 (2003)

S6. S. Hughes, A. Jagannath, D. Hickey, S. Gatti, M. Wood, S. N. Peirson, R. G. Foster, M. W. Hankins, Using siRNA to define functional interactions between melanopsin and multiple G Protein partners. *Cell Mol Life Sci*, (2014)

S7. A. Jagannath, R. Butler, S. I. Godinho, Y. Couch, L. A. Brown, S. R. Vasudevan, K. C. Flanagan, D. Anthony, G. C. Churchill, M. J. Wood, G. Steiner, M. Ebeling, M. Hossbach, J. G. Wettstein, G. E. Duffield, S. Gatti, M. W. Hankins, R. G. Foster, S. N. Peirson, The CRTC1-SIK1 pathway regulates entrainment of the circadian clock. *Cell* **154**, 1100-1111 (2013)

S8. S. S. Pires, S. Hughes, M. Turton, Z. Melyan, S. N. Peirson, L. Zheng, M. Kosmaoglou, J. Bellingham, M. E. Cheetham, R. J. Lucas, R. G. Foster, M. W. Hankins, S. Halford, Differential expression of two

distinct functional isoforms of melanopsin (Opn4) in the mammalian retina. *J Neurosci* **29**, 12332-12342 (2009)

S9. S. Hughes, T. S. Watson, S. N. Peirson, R. G. Foster, M. W. Hankins, Non-uniform distribution and spectral tuning of photosensitive retinal ganglion cells of the mouse retina. *Curr Biol* **Under Review**, (2013)
