# Supplementary material for: Isoforms of Melanopsin Mediate Different Behavioral Responses to Light
Source: Curr Biol. 2015 Sep 21;25(18):2430–4. doi: 10.1016/j.cub.2015.07.071 (PMC4580334; doi:10.1016/j.cub.2015.07.071)
Supplement: Document S2. Article plus Supplemental Information [file mmc2.pdf]

Report

Current Biology

Isoforms of Melanopsin Mediate Different Behavioral Responses to Light

Highlights

- The retinal photopigment melanopsin is alternatively spliced
- The isoforms mediate different physiological and behavioral responses to light
- The short variant regulates pupil size, the long, negative masking of activity
- Both variants regulate sleep and phase shifting of circadian rhythms

Authors

Aarti Jagannath, Steven Hughes, Amr Abdelgany, ..., Matthew J.A. Wood, Russell G. Foster, Stuart N. Peirson

Correspondence

russell.foster@eye.ox.ac.uk (R.G.F.),
stuart.peirson@eye.ox.ac.uk (S.N.P.)

In Brief

We show that the splice variants of the retinal photopigment melanopsin regulate different non-image-forming responses to light, including, for example, pupil constriction, which is mediated by the short variant alone. Our findings demonstrate that splice variants of a single receptor gene can regulate strikingly different behaviors.

Accession Numbers

KT381095

Jagannath et al., 2015, Current Biology *25*, 2430–2434
September 21, 2015 ©2015 The Authors
http://dx.doi.org/10.1016/j.cub.2015.07.071

Current Biology
Report

Isoforms of Melanopsin Mediate Different Behavioral Responses to Light

Aarti Jagannath,[1,2,5] Steven Hughes,[1,2,5] Amr Abdelgany,[3] Carina A. Pothecary,[1] Simona Di Pretoro,[1] Susana S. Pires,[1] Athanasios Vachtsevanos,[1] Violetta Pilorz,[1] Laurence A. Brown,[1] Markus Hossbach,[4] Robert E. MacLaren,[1] Stephanie Halford,[1] Silvia Gatti,[2] Mark W. Hankins,[1] Matthew J.A. Wood,[3] Russell G. Foster,[1,*] and Stuart N. Peirson[1,*]
[1]Nuffield Laboratory of Ophthalmology, John Radcliffe Hospital, University of Oxford, Levels 5-6 West Wing, Headley Way, Oxford OX3 9DU, UK
[2]F. Hoffmann-La Roche AG, Pharma Research and Early Development, DTA Neuroscience pRED, Grenzacherstrasse 124, Basel 4070, Switzerland
[3]Department of Physiology, Anatomy and Genetics, South Parks Road, Oxford OX1 3QX, UK
[4]Axolabs GmbH, Fritz-Hornschuch-Straße 9, 95326 Kulmbach, Germany
[5]Co-first author
*Correspondence: russell.foster@eye.ox.ac.uk (R.G.F.), stuart.peirson@eye.ox.ac.uk (S.N.P.)
http://dx.doi.org/10.1016/j.cub.2015.07.071
This is an open access article under the CC BY license (http://creativecommons.org/licenses/by/4.0/).

SUMMARY

Melanopsin (OPN4) is a retinal photopigment that mediates a wide range of non-image-forming (NIF) responses to light [1, 2] including circadian entrainment [3], sleep induction [4], the pupillary light response (PLR) [5], and negative masking of locomotor behavior (the acute suppression of activity in response to light) [6]. How these diverse NIF responses can all be mediated by a single photopigment has remained a mystery. We reasoned that the alternative splicing of melanopsin could provide the basis for functionally distinct photopigments arising from a single gene. The murine melanopsin gene is indeed alternatively spliced, producing two distinct isoforms, a short (OPN4S) and a long (OPN4L) isoform, which differ only in their C terminus tails [7]. Significantly, both isoforms form fully functional photopigments [7]. Here, we show that different isoforms of OPN4 mediate different behavioral responses to light. By using RNAi-mediated silencing of each isoform in vivo, we demonstrated that the short isoform (OPN4S) mediates light-induced pupillary constriction, the long isoform (OPN4L) regulates negative masking, and both isoforms contribute to phase-shifting circadian rhythms of locomotor behavior and light-mediated sleep induction. These findings demonstrate that splice variants of a single receptor gene can regulate strikingly different behaviors.

RESULTS AND DISCUSSION

To ensure that the splice variants seen in murine melanopsin are not unique to this species [7], we sought confirmation for the existence of *Opn4* isoforms in other mammals. We found empirical evidence for *Opn4S* and *Opn4L* variants in humans (Figure S1), and bioinformatic analysis of genomic sequences indicates the presence of similar open reading frames in several other mammalian species including the chimpanzee *Pan troglodytes* and the opossum *Monodelphis domestica* (data not shown). Non-mammalian species have also been shown to possess multiple genes and splice variants of melanopsin, including chicken [8], *Xenopus*, and elephant shark [9]. Such findings argue that the splice variants of OPN4 in mice are unlikely to be unique to this species but are of functional significance across the vertebrates.

RNAi provides an acute and exquisitely specific tool to dissect the role of the different melanopsin isoforms in vivo [10]. We designed and tested siRNAs against each isoform of *Opn4* as well as a universal sequence that silenced both isoforms. We confirmed the successful delivery of siRNA to pRGCs, along with their efficacy and specificity both in vivo and in vitro (Figure S2). Following delivery, we studied the pupillary light response (PLR), negative masking, phase shifting of circadian rhythms of locomotor behavior, and light-induced sleep induction after silencing of both or either *Opn4* isoform in vivo.

Melanopsin-deficient ($Opn4^{-/-}$) mice show an attenuated PLR [5], and mice lacking rods, cones, and pRGCs ($Gnat1^{-/-}$, $Cnga3^{-/-}$, and $Opn4^{-/-}$) show no PLR [2]. In *rd/rd cl* mice, which lack rods and cones, siRNA-mediated knockdown of *Opn4* should result in a substantially reduced PLR [10], as OPN4 is the only remaining photopigment [1]. We would not predict the complete loss of the PLR as siRNA knockdown is not complete in vivo (Figure S2) [11–14]. We found that knockdown of *Opn4* in the eye did indeed severely attenuate pupil constriction in response to light in the contralateral eye (Figure 1). Knockdown of *Opn4S* also resulted in a significant attenuation of the PLR, whereas knockdown of *Opn4L* had no effect (Figure 1), indicating that OPN4S provides the primary input of the light response driving the PLR.

Phase shifting of circadian rhythms in response to a nocturnal light pulse has been shown previously to be attenuated in $Opn4^{-/-}$ mice [3]. siRNA was administered bilaterally to achieve knockdown of either or both isoforms of *Opn4* in both eyes. Four days later, the animals received a 30 min light pulse at circadian time (CT) 16. *Opn4* knockdown resulted in smaller phase delays compared with the control injected with a non-targeting siRNA (reduced to 50%). Knockdown of either isoform caused a modest

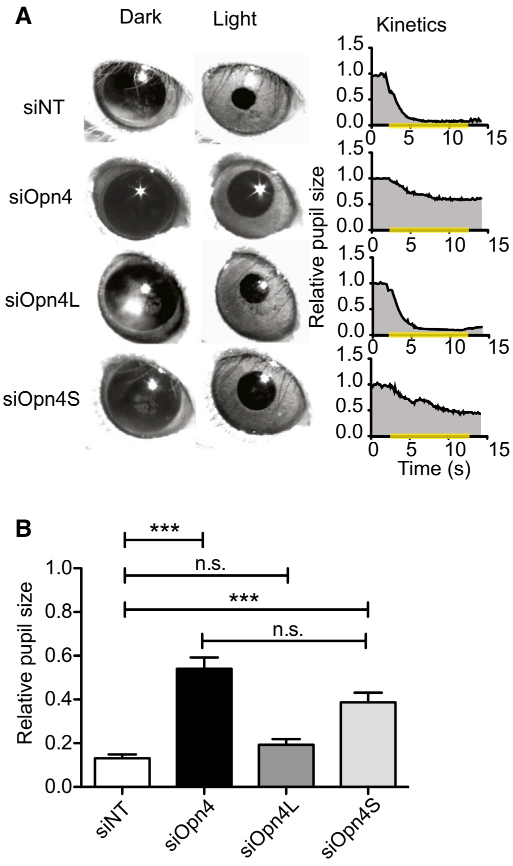

Figure 1. OPN4S Mediates the Pupillary Light Response

Animals received siOpn4, siOpn4L, or siOpn4S in the one eye and siNT in the contralateral eye. The left eye was then stimulated by exposure to bright 480 nm light, and pupil constriction was imaged and measured from the right eye.

(A) Images of pupil before (left panel) and immediately after (right panel) exposure to light for 10 s. Graph on right shows kinetics of pupil constriction, with pupil size normalized to dark level on the y axis. The yellow bar indicates duration of light exposure. Representative images and graphs for animals injected with siNT, siOpn4, siOpn4L, and siOpn4S are included.

(B) Average pupil constriction at the end of the light pulse for animals injected with siRNA as indicated on the y axis, showing significantly attenuated pupil constriction for siOpn4 (0.58 ± 0.06 versus 0.13 ± 0.017; n = 7; p = 0.0001) and siOpn4S (0.39 ± 0.04; n = 10; p = 0.0002). siOpn4L did not significantly attenuate pupil constriction (0.19 ± 0.03; n = 10; p = 0.09), and siOpn4S treatment did not statistically differ from siOpn4 treatment (p = 0.08). *p < 0.05, **p < 0.01, ***p < 0.001; one-way ANOVA with Tukey's post-tests. Error bars represent the SEM.

See also Figures S1, S2, and S4.

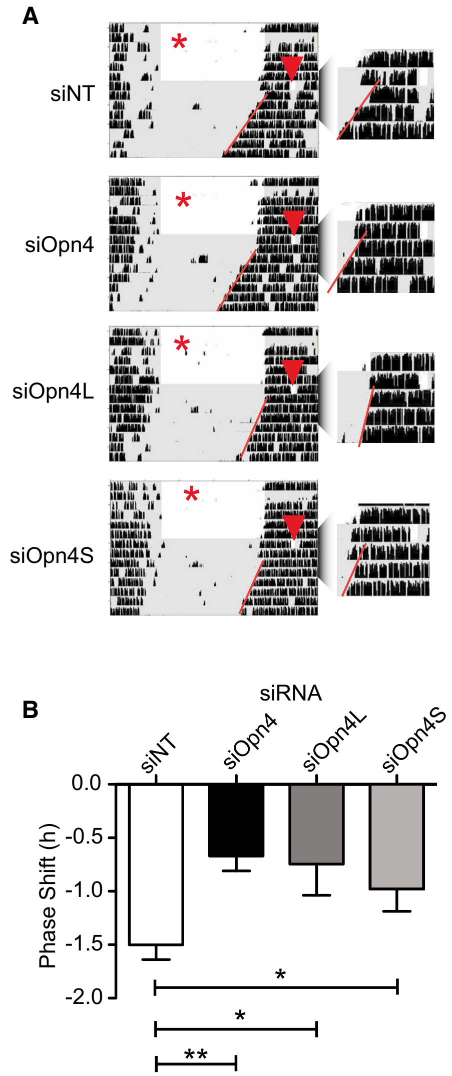

Figure 2. Both Isoforms of OPN4 Mediate Phase Shifting of Circadian Rhythms

(A) Representative actograms from animals given intravitreal bilateral injection (indicated by a red star) of siNT, siOpn4, siOpn4S, and siOpn4L. Significantly reduced phase shifting after a 30 min CT16 light pulse (indicated by a red arrow) is seen for all three *Opn4*-targeting siRNAs versus siNT control. Actograms are enlarged around the light pulse for clarity.

(B) Histogram showing average phase shift for siOpn4-treated animals (−0.68 ± 0.13 versus −1.50 ± 0.14 hr; n = 12; p = 0.0003) and siOpn4L and siOpn4S (−0.74 ± 0.29; p = 0.01; n = 8 and −0.98 ± 0.21; p = 0.04; n = 8, respectively). *p < 0.05, **p < 0.01; one-way ANOVA with Tukey's post-tests. Error bars represent the SEM.

See also Figures S1 and S2.

but significant reduction in the magnitude of phase shifts (Figure 2). These data indicate that light responses from both isoforms reach the SCN and mediate phase-shifting responses.

We then proceeded to evaluate the effect of siRNA-mediated knockdown of *Opn4* on negative masking, another response that has been shown to be attenuated in *Opn4*$^{-/-}$ mice [6]. After bilateral knockdown of *Opn4* or its isoforms, animals were given a 10 min light pulse at half an hour after lights off (ZT 12.5) every day, which was designed to avoid the phase-shifting effects of light. Knockdown of both isoforms resulted in attenuated negative masking during the 10 min light pulse, similar to that seen in *Opn4*$^{-/-}$ mice [6] (Figures 3A and 3B). Knockdown of *Opn4S* had no significant effect on negative masking, whereas *Opn4L* silencing attenuated this response, to the same degree as knockdown of *Opn4* itself (siOpn4L = 75 ± 14; siOpn4 = 62 ± 8; p = 0.36; Figures 3A, 3C, and S3). These results led us to conclude that light signaling by the long isoform of OPN4 is responsible for negative masking.

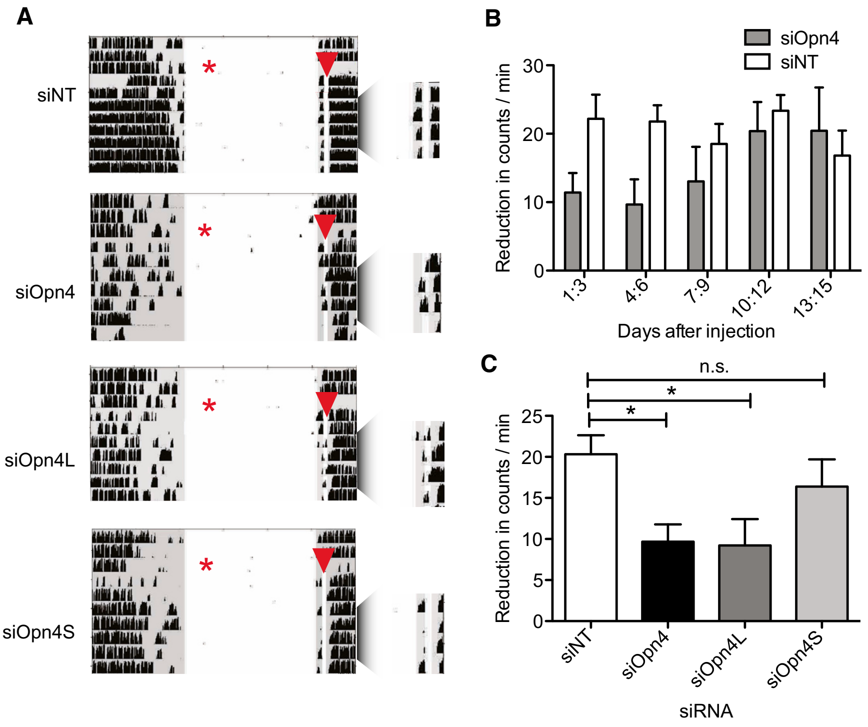

Figure 3. OPN4L Mediates Negative Masking

(A) Representative actograms from animals given intravitreal bilateral injection (indicated by a red star) of siNT, siOpn4, siOpn4S, and siOpn4L show negative masking after a 10 min ZT12.5 light pulse (indicated by a red arrow) every day following injection. This protocol avoids significant light-induced phase shifts. Negative masking is attenuated with siOpn4 and siOpn4L. Actograms are enlarged around light pulse for clarity.

(B) Histogram of reduction in activity during the light pulse (as compared with activity preceding the light pulse) across 3-day bins as indicated on the y axis.

(C) Histogram of reduction in average (of days 2–8) wheel-running activity during the nocturnal light pulse shows smaller reductions in activity with siOpn4 (11.1 ± 1.6 versus 22.2 ± 2.3 siNT) and siOpn4L-treated animals (9.7 ± 3.6). siOpn4S had no significant effect (18.0 ± 3.4). n = 6. $^*p < 0.05$; one-way ANOVA with Dunnett's post-test. Error bars represent the SEM.

See also Figures S1–S4.

Our studies [4], and those of others [15–17], have shown OPN4-mediated signaling plays an important role in the regulation of sleep-wake states. pRGCs can regulate this response via a direct but sparse innervation to the ventrolateral preoptic nuclei [18, 19] (VLPO; the sleep switch) or via a substantial relay system arising from the SCN consisting of the vSPZ (sub-paraventricular zone) and the DMH (dorsomedial hypothalamus) [20]. In view of the findings presented in Figure 2 that both OPN4L and OPN4S signal light to the SCN and that light via the SCN plays an important role in sleep regulation, we predicted that both splice variants would be important in regulating light-induced sleep. This prediction proved correct. Bilateral knockdown for *Opn4* and its isoforms was undertaken. Four days later, mice were exposed to a 1 hr light pulse at ZT 14, during which sleep was measured using video monitoring [21]. Simultaneous passive infrared recordings were made to assess activity levels. Knockdown of both isoforms results in attenuated induction of sleep, with knockdown animals showing higher locomotor activity (Figure 4A) and a 50% reduction in sleep during the 1 hr light pulse compared with the control (Figure 4B). Knockdown of either *Opn4S* or *Opn4L* also reduced the levels of sleep (Figure 4B). Activity in the *Opn4* knockdown animals was much higher than the control, as expected, and this was also seen after *Opn4L* knockdown (Figures 4A, 4C, and S3), consistent with the negative masking results described in Figure 3. Animals with knockdown of *Opn4S* show attenuated activity during the light pulse (Figures 4A and 4C), although the animals showed reduced sleep, showing that strong light-aversive responses [22] remained in these animals whereas the propensity to sleep was attenuated.

Recent work has demonstrated a role for PLCB4 (1-phosphatidylinositol-4,5-bisphosphate phosphodiesterase beta-4) in the melanopsin-signaling cascade [23], presumably acting downstream of Gnaq/11 type G proteins [24]. To determine whether both isoforms signal via the same cascade, we undertook *Plcb4* silencing (Figure S4A). Silencing attenuated the PLR (Figures S4B and S4C) and also negative masking (Figures S4D and S4E), mirroring the effect of *Opn4* silencing, indicating that PLCB4 participates in the signaling cascade of both OPN4S and OPN4L isoforms.

Here, we show that OPN4 isoforms mediate different behavioral responses to light, such that OPN4S mediates the PLR; OPN4L negative masking and both isoforms mediate phase shifting of locomotor behavior and sleep induction in response to light. Further, both isoforms signal via PLCB4. OPN4L and OPN4S differ only at their C-terminal tails, and thus, it seems likely that any functional differences between these isoforms must reside within these regions [7]. Bioinformatic analysis indicates that the longer tail of OPN4L may contain additional phosphorylation sites, and these may confer functional differences in responses mediated by OPN4L and OPN4S, most likely influencing rates of adaptation, recovery, and sensitization, as has been shown for other G-protein-coupled receptors, where phosphorylation of the C-terminal tail can significantly change receptor signaling [25, 26].

To date, at least five distinct subtypes of pRGC have been described, termed M1–M5 [27, 28]. These cell types project to different regions of the brain [28] and exhibit light responses with markedly different kinetics [29]. However, whereas it may be logical to conclude that different pRGC subtypes mediate different non-image-forming (NIF) responses to light on the basis of their anatomical projections [27], empirical evidence is largely lacking. Indeed, the only study to show a direct link between pRGC subtype and behavior is from Chen et al. [30]. Specifically, they showed that a subpopulation of *Brn3b*-negative M1 pRGCs that project to the SCN are capable of driving circadian

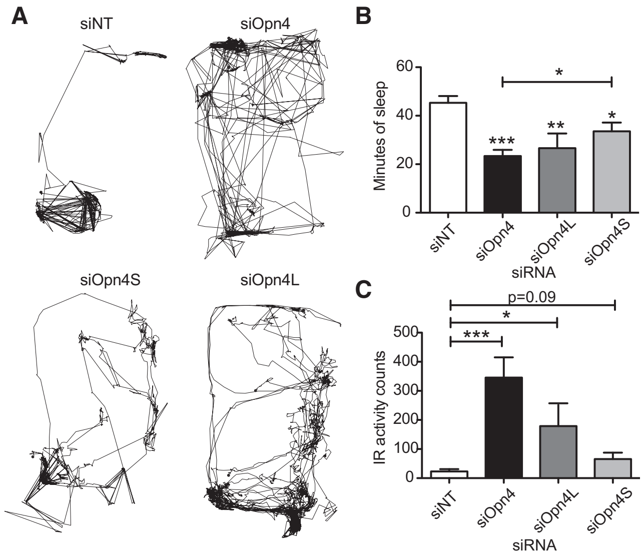

Figure 4. Both Isoforms of OPN4 Mediate Sleep Induction

(A) Animals were given bilateral siRNA (siNT, siOpn4, siOpn4S, and siOpn4L) injections and, 4 days later, given a 1 hr light pulse at ZT14, during which videos were recorded and analyzed. The traces show activity patterns during the light pulse of individually housed mice receiving siRNAs as indicated. siNT-injected animals restricted their movements to their nest, where they spent the majority of time sleeping. The *Opn4* knockdown animals showed markedly decreased levels of sleep.

(B) Total sleep levels (measured as bouts of >40 s immobility) were measured [21] during the course of the light pulse. *Opn4* knockdown animals show severely attenuated sleep induction during the light pulse (23.38 ± 2.5 min versus 45.34 ± 2.8 min; n = 10; $p < 0.0001$). *Opn4L* and *Opn4S* knockdown animals also showed reduced levels of sleep (26.65 ± 6.0; n = 10; $p < 0.01$ and 33.58 ± 3.6; n = 10; $p = 0.02$).

(C) Locomotor activity during the light pulse as measured via passive infrared recordings (PIR) for the same animals as above showing attenuated reductions in the case of *Opn4* and *Opn4L* knockdown (95.4% ± 30.7% and 35.4% ± 11.9% versus 7.4% ± 4.1% for siNT, respectively). Locomotor activity as measured by PIR during the first 10 min of the light pulse to compare with Figure 3 are provided in Figure S3C, and activity during the hour preceding the light pulse shows no significant differences across the groups as indicated in Figure S3D. *$p < 0.05$, **$p < 0.01$, ***$p < 0.001$; one-way ANOVA with Tukey's post-tests. Error bars represent the SEM.

See also Figures S1–S3.

entrainment following the ablation of all other pRGCs (*Brn3b*-positive and including M1–M5 pRGCs) [30]. Ablation of *Brn3b*-positive M1–M5 pRGCs was shown to disrupt the PLR, yet given the widespread loss of pRGC subtypes using this approach, and the complete loss of OPN innervations, it is not possible to conclude from this study which class of pRGC mediates the PLR or other NIF responses to light.

An intuitive explanation for the differences we describe would be the differential expression of OPN4L and OPN4S isoforms in different pRGC subtypes. However, whereas this may provide a partial explanation for our findings, it cannot provide the complete answer. For example, here, we show that the PLR is mediated by OPN4S, which is only expressed in M1 pRGCs [7]. M1 cells co-express OPN4L and OPN4S [7], and we show that silencing *Opn4L* produces no significant change in PLR (Figure 1). Thus, for pupil constriction, OPN4L cannot compensate for the loss of OPN4S in M1 pRGCs. We have shown that Opn4L is expressed at much-lower levels in the retina (about 40-fold less than *Opn4S*) [7]. Whereas silencing *Opn4L* would specifically target M2 cells (which express just *Opn4L*), it may have little effect on M1 cells, which will still express high levels of *Opn4S*. It is entirely plausible that this may be sufficient to drive a pupillary response. In addition, it is also possible that the two isoforms dimerize/oligomerize in different combinations, and these hetero/homo oligomers have different signaling properties. This has been demonstrated amply with several other GPCRs (see Palczewski et al., 2010 [31] for review on rhodopsin oligomerization). Hetero oligomerization of GPCRs can result in differences in pharmacology and downstream signaling pathways and also modulate the strength of the signal. For example, heterodimerization of the opioid δ/κ receptors results in signaling potentiation [32] and heterodimerization is required for transactivation of the GABA receptors GB1 and GB2 [33].

Collectively, our findings demonstrate that splice variants of the melanopsin gene can regulate strikingly different behaviors. In addition to highlighting the diversity of melanopsin signaling, these data provide one of the very few examples we have across the animal kingdom that isoforms of a single gene can regulate highly divergent behaviors. Furthermore, this is the only example in visual biology that naturally occurring opsin isoforms mediate different physiological and behavioral responses to light.

EXPERIMENTAL PROCEDURES

All animals used were retinal degenerate *rd/rd* (C3H/HeN; Harlan UK) mice (older than 80 days) lacking rod and the majority of cone photoreceptors, unless otherwise indicated as *rd/rd cl* [1]. All animals were housed under a 12:12 LD cycle with food and water ad libitum. All procedures were conducted in accordance with the Animals (Scientific Procedures) Act 1986 and the University of Oxford Policy on the Use of Animals in Scientific Research (PPL 70/6382 and 30/2812). All procedures were reviewed by the Clinical Medicine Animal Welfare and Ethical Review Body (AWERB). Animals were sacrificed via schedule 1 methods in accordance with the UK Home Office Animals (Scientific Procedures) Act 1986.

ACCESSION NUMBERS

The accession number for the human Opn4L sequence reported in this paper is GenBank: KT381095.

SUPPLEMENTAL INFORMATION

Supplemental Information includes Supplemental Experimental Procedures and four figures and can be found with this article online at http://dx.doi.org/10.1016/j.cub.2015.07.071.

AUTHOR CONTRIBUTIONS

A.J. performed all in vivo RNAi experiments and wrote the manuscript with input from S. Hughes, R.G.F., and S.N.P. S. Hughes performed the immunohistochemistry and initial in vitro RNAi experiments. A.A. designed the isoform-specific siRNAs with input from M.J.A.W. M.H. designed and provided siRNAs suitable for in vivo use. C.A.P., S.S.P., V.P., L.A.B., A.V., and R.E.M. assisted with in vivo experiments. S.S.P. and S. Halford performed the studies with human OPN4 isoforms. S.G., M.W.H., M.J.A.W., R.G.F., and S.N.P. provided input into the conception of the project and all aspects of experimental work. S.N.P. and R.G.F. reviewed and edited the manuscript.

CellPress

ACKNOWLEDGMENTS

We thank Professor Rob Lucas (University of Manchester) for useful discussions. Funding is from the Wellcome Trust (090684/Z/09/Z and 098461/Z/12/Z), BBSRC (BB/I021086/1), and F. Hoffmann-La Roche.

Received: December 22, 2014
Revised: May 18, 2015
Accepted: July 30, 2015
Published: August 27, 2015

REFERENCES

1. Freedman, M.S., Lucas, R.J., Soni, B., von Schantz, M., Muñoz, M., David-Gray, Z., and Foster, R. (1999). Regulation of mammalian circadian behavior by non-rod, non-cone, ocular photoreceptors. Science *284*, 502–504.
2. Hattar, S., Lucas, R.J., Mrosovsky, N., Thompson, S., Douglas, R.H., Hankins, M.W., Lem, J., Biel, M., Hofmann, F., Foster, R.G., and Yau, K.W. (2003). Melanopsin and rod-cone photoreceptive systems account for all major accessory visual functions in mice. Nature *424*, 76–81.
3. Panda, S., Sato, T.K., Castrucci, A.M., Rollag, M.D., DeGrip, W.J., Hogenesch, J.B., Provencio, I., and Kay, S.A. (2002). Melanopsin (Opn4) requirement for normal light-induced circadian phase shifting. Science *298*, 2213–2216.
4. Lupi, D., Oster, H., Thompson, S., and Foster, R.G. (2008). The acute light-induction of sleep is mediated by OPN4-based photoreception. Nat. Neurosci. *11*, 1068–1073.
5. Lucas, R.J., Hattar, S., Takao, M., Berson, D.M., Foster, R.G., and Yau, K.W. (2003). Diminished pupillary light reflex at high irradiances in melanopsin-knockout mice. Science *299*, 245–247.
6. Mrosovsky, N., and Hattar, S. (2003). Impaired masking responses to light in melanopsin-knockout mice. Chronobiol. Int. *20*, 989–999.
7. Pires, S.S., Hughes, S., Turton, M., Melyan, Z., Peirson, S.N., Zheng, L., Kosmaoglou, M., Bellingham, J., Cheetham, M.E., Lucas, R.J., et al. (2009). Differential expression of two distinct functional isoforms of melanopsin (Opn4) in the mammalian retina. J. Neurosci. *29*, 12332–12342.
8. Torii, M., Kojima, D., Okano, T., Nakamura, A., Terakita, A., Shichida, Y., Wada, A., and Fukada, Y. (2007). Two isoforms of chicken melanopsins show blue light sensitivity. FEBS Lett. *581*, 5327–5331.
9. Davies, W.I., Tay, B.H., Zheng, L., Danks, J.A., Brenner, S., Foster, R.G., Collin, S.P., Hankins, M.W., Venkatesh, B., and Hunt, D.M. (2012). Evolution and functional characterisation of melanopsins in a deep-sea chimaera (elephant shark, Callorhinchus milii). PLoS ONE *7*, e51276.
10. Hughes, S., Jagannath, A., Hickey, D., Gatti, S., Wood, M., Peirson, S.N., Foster, R.G., and Hankins, M.W. (2015). Using siRNA to define functional interactions between melanopsin and multiple G Protein partners. Cell. Mol. Life Sci. *72*, 165–179.
11. Jagannath, A., Butler, R., Godinho, S.I., Couch, Y., Brown, L.A., Vasudevan, S.R., Flanagan, K.C., Anthony, D., Churchill, G.C., Wood, M.J., et al. (2013). The CRTC1-SIK1 pathway regulates entrainment of the circadian clock. Cell *154*, 1100–1111.
12. Kim, S.S., Wang, H., Li, X.Y., Chen, T., Mercaldo, V., Descalzi, G., Wu, L.J., and Zhuo, M. (2011). Neurabin in the anterior cingulate cortex regulates anxiety-like behavior in adult mice. Mol. Brain *4*, 6.
13. Meacham, C.E., Ho, E.E., Dubrovsky, E., Gertler, F.B., and Hemann, M.T. (2009). In vivo RNAi screening identifies regulators of actin dynamics as key determinants of lymphoma progression. Nat. Genet. *41*, 1133–1137.
14. June, H.L., Liu, J., Warnock, K.T., Bell, K.A., Balan, I., Bollino, D., Puche, A., and Aurelian, L. (2015). CRF-amplified neuronal TLR4/MCP-1 signaling regulates alcohol self-administration. Neuropsychopharmacology *40*, 1549–1559.
15. Hubbard, J., Ruppert, E., Gropp, C.M., and Bourgin, P. (2013). Non-circadian direct effects of light on sleep and alertness: lessons from transgenic mouse models. Sleep Med. Rev. *17*, 445–452.
16. Altimus, C.M., Güler, A.D., Villa, K.L., McNeill, D.S., Legates, T.A., and Hattar, S. (2008). Rods-cones and melanopsin detect light and dark to modulate sleep independent of image formation. Proc. Natl. Acad. Sci. USA *105*, 19998–20003.
17. Tsai, J.W., Hannibal, J., Hagiwara, G., Colas, D., Ruppert, E., Ruby, N.F., Heller, H.C., Franken, P., and Bourgin, P. (2009). Melanopsin as a sleep modulator: circadian gating of the direct effects of light on sleep and altered sleep homeostasis in Opn4(-/-) mice. PLoS Biol. *7*, e1000125.
18. Gooley, J.J., Lu, J., Fischer, D., and Saper, C.B. (2003). A broad role for melanopsin in nonvisual photoreception. J. Neurosci. *23*, 7093–7106.
19. Hattar, S., Kumar, M., Park, A., Tong, P., Tung, J., Yau, K.W., and Berson, D.M. (2006). Central projections of melanopsin-expressing retinal ganglion cells in the mouse. J. Comp. Neurol. *497*, 326–349.
20. Chou, T.C., Scammell, T.E., Gooley, J.J., Gaus, S.E., Saper, C.B., and Lu, J. (2003). Critical role of dorsomedial hypothalamic nucleus in a wide range of behavioral circadian rhythms. J. Neurosci. *23*, 10691–10702.
21. Fisher, S.P., Godinho, S.I., Pothecary, C.A., Hankins, M.W., Foster, R.G., and Peirson, S.N. (2012). Rapid assessment of sleep-wake behavior in mice. J. Biol. Rhythms *27*, 48–58.
22. Semo, M., Gias, C., Ahmado, A., Sugano, E., Allen, A.E., Lawrence, J.M., Tomita, H., Coffey, P.J., and Vugler, A.A. (2010). Dissecting a role for melanopsin in behavioural light aversion reveals a response independent of conventional photoreception. PLoS ONE *5*, e15009.
23. Xue, T., Do, M.T., Riccio, A., Jiang, Z., Hsieh, J., Wang, H.C., Merbs, S.L., Welsbie, D.S., Yoshioka, T., Weissgerber, P., et al. (2011). Melanopsin signalling in mammalian iris and retina. Nature *479*, 67–73.
24. Hankins, M.W., Peirson, S.N., and Foster, R.G. (2008). Melanopsin: an exciting photopigment. Trends Neurosci. *31*, 27–36.
25. Krupnick, J.G., and Benovic, J.L. (1998). The role of receptor kinases and arrestins in G protein-coupled receptor regulation. Annu. Rev. Pharmacol. Toxicol. *38*, 289–319.
26. Ritter, S.L., and Hall, R.A. (2009). Fine-tuning of GPCR activity by receptor-interacting proteins. Nat. Rev. Mol. Cell Biol. *10*, 819–830.
27. Schmidt, T.M., Chen, S.K., and Hattar, S. (2011). Intrinsically photosensitive retinal ganglion cells: many subtypes, diverse functions. Trends Neurosci. *34*, 572–580.
28. Ecker, J.L., Dumitrescu, O.N., Wong, K.Y., Alam, N.M., Chen, S.K., LeGates, T., Renna, J.M., Prusky, G.T., Berson, D.M., and Hattar, S. (2010). Melanopsin-expressing retinal ganglion-cell photoreceptors: cellular diversity and role in pattern vision. Neuron *67*, 49–60.
29. Schmidt, T.M., and Kofuji, P. (2009). Functional and morphological differences among intrinsically photosensitive retinal ganglion cells. J. Neurosci. *29*, 476–482.
30. Chen, S.K., Badea, T.C., and Hattar, S. (2011). Photoentrainment and pupillary light reflex are mediated by distinct populations of ipRGCs. Nature *476*, 92–95.
31. Palczewski, K. (2010). Oligomeric forms of G protein-coupled receptors (GPCRs). Trends Biochem. Sci. *35*, 595–600.
32. Jordan, B.A., and Devi, L.A. (1999). G-protein-coupled receptor heterodimerization modulates receptor function. Nature *399*, 697–700.
33. Galvez, T., Duthey, B., Kniazeff, J., Blahos, J., Rovelli, G., Bettler, B., Prézeau, L., and Pin, J.P. (2001). Allosteric interactions between GB1 and GB2 subunits are required for optimal GABA(B) receptor function. EMBO J. *20*, 2152–2159.

Current Biology

Supplemental Information

Isoforms of Melanopsin Mediate Different Behavioral Responses to Light

Aarti Jagannath, Steven Hughes, Amr Abdelgany, Carina A. Pothecary, Simona Di Pretoro, Susana S. Pires, Athanasios Vachtsevanos, Violetta Pilorz, Laurence A. Brown, Markus Hossbach, Robert E. MacLaren, Stephanie Halford, Silvia Gatti, Mark W. Hankins, Matthew J.A. Wood, Russell G. Foster, and Stuart N. Peirson

Figure S1

A

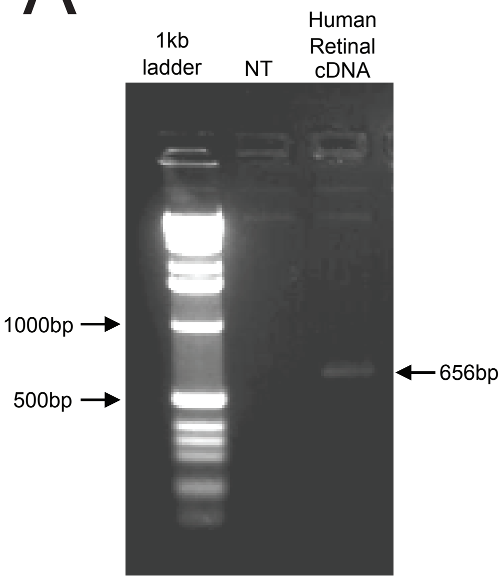

B

```
HOPN47F                                 Exon7
GCCAAGGCCTCTGCAATCCACAACCCCATCATTTACGCCATCACCCACCCCAAGTACAGG   60
A  K  A  S  A  I  H  N  P  I  I  Y  A  I  T  H  P  K  Y  R
GTGGCCATTGCCCAGCACCTGCCCTGCCTGGGGGTGCTGCTGGGTGTATCACGCCGGCAC  120
V  A  I  A  Q  H  L  P  C  L  G  V  L  L  G  V  S  R  R  H
                                    Exon8
AGTCGCCCCTACCCCAGCTACCGCTCCACCCACCGCTCCACGCTGACCAGCCACACCTCC  180
S  R  P  Y  P  S  Y  R  S  T  H  R  S  T  L  T  S  H  T  S
AACCTCAGCTGGATCTCCATACGGAGGCGCCAGGAGTCCCTGGGCTCGGAGAGTGAGGTG  240
N  L  S  W  I  S  I  R  R  R  Q  E  S  L  G  S  E  S  E  V
                                    Exon9
GGCTGGACACACATGGAGGCAGCAGCTGTGTGGGGAGCTGCCCAGCAAGCAAATGGGCGG  300
G  W  T  H  M  E  A  A  A  V  W  G  A  A  Q  Q  A  N  G  R
TCCCTCTACGGTCAGGGTCTGGAGGACTTGGAAGCCAAGGCACCCCCCAGACCCCAGGGA  360
S  L  Y  G  Q  G  L  E  D  L  E  A  K  A  P  P  R  P  Q  G
CACGAAGCAGAGACTCCAGGGAAGGTGACTGGGCCCGGTACCTGCCAATCCACAAAGGGG  420
H  E  A  E  T  P  G  K  V  T  G  P  G  T  C  Q  S  T  K  G
TGGGGGTTAGGGGCCAGTAGCCCAGGGAGAGGCCAGGAAAGAGAGACTTGTTCTCATGGG  480
W  G  L  G  A  S  S  P  G  R  G  Q  E  R  E  T  C  S  H  G
CAGGGGCGATATCCTCCAGGAATCACCTGCTCCCAGCACCTCCCAGCTTTTCCTTGTCTG  540
Q  G  R  Y  P  P  G  I  T  C  S  Q  H  L  P  A  F  P  C  L
CCTGGGGTTCTCTGTGACTCTGAGATGCTCTGAgcagggcctgcatatggtctgcacatg  600
P  G  V  L  C  D  S  E  M  L  *
tgtgtgtgtgtgtgtgatcatgcaactctgactctgcatggtgctgttggctgtgc      656
                                           HOPN4Long
```

C

```
HumanLong     AKASAIHNPIIYAITHPKYRVAIAQHLPCLGVLLGVSRRHSRPYPSYRSTHRSTLTSHTS 60
MouseLong     AKASAIHNPIIYAITHPKYRVAIAQHLPCLGVLLGVSGQRSHPSLSYRSTHRSTLSSQSS 60
HumanShort    AKASAIHNPIIYAITHPKYRVAIAQHLPCLGVLLGVSRRHSRPYPSYRSTHRSTLTSHTS 60
MouseShort    AKASAIHNPIIYAITHPKYRVAIAQHLPCLGVLLGVSGQRSHPSLSYRSTHRSTLSSQSS 60
              ************************************* ::*:*  **********:*::*!

HumanLong     NLSWISIRRRQESLGSESEVGWTHMEAAAVWGAAQQANGRSLYGQGLEDLEAKAPPRPQG 120
MouseLong     DLSWISGRKRQESLGSESEVGWTDTETTAAWGAAQQASGQSFCSQNLEDGELKASSSPQV 120
HumanShort    NLSWISIRRRQESLGSESEVGWTHMEAAAVWGAAQQANGRSLYGQGLEDLEAKAPPRPQG 120
MouseShort    DLSWISGRKRQESLGSESEVGWTDTETTAAWGAAQQASGQSFCSQNLEDGELKASSSPQV 120
              :***** *:**************. *::*.*******.*:*: .*.*** * **.. **

HumanLong     HEAETPGKVTGPGTCQSTKGWGLGASSPGRGQE-RETCSHGQGRYPPGITCSQHLPAFPC 179
MouseLong     QRSKTP-KVPGPSTCRPMKGQGARPSSLRGDQKGRLAVCTGLSECPHPHT-SQFPLAF-- 176
              :.::** **.**.**:. ** *  .**   .*: * : . * .. *   * **.  **
HumanShort    HEAETPGKTKGLIPSQDPRM                                         140
MouseShort    QRSKTP-KTKGHLPSLDLGM                                         139
              :.::** **** :** *  *

HumanLong     LPGVLCDSEML                                                  190
MouseLong     LEDDVTLRH-L                                                  186
              * . :   . *
```

Figure S2

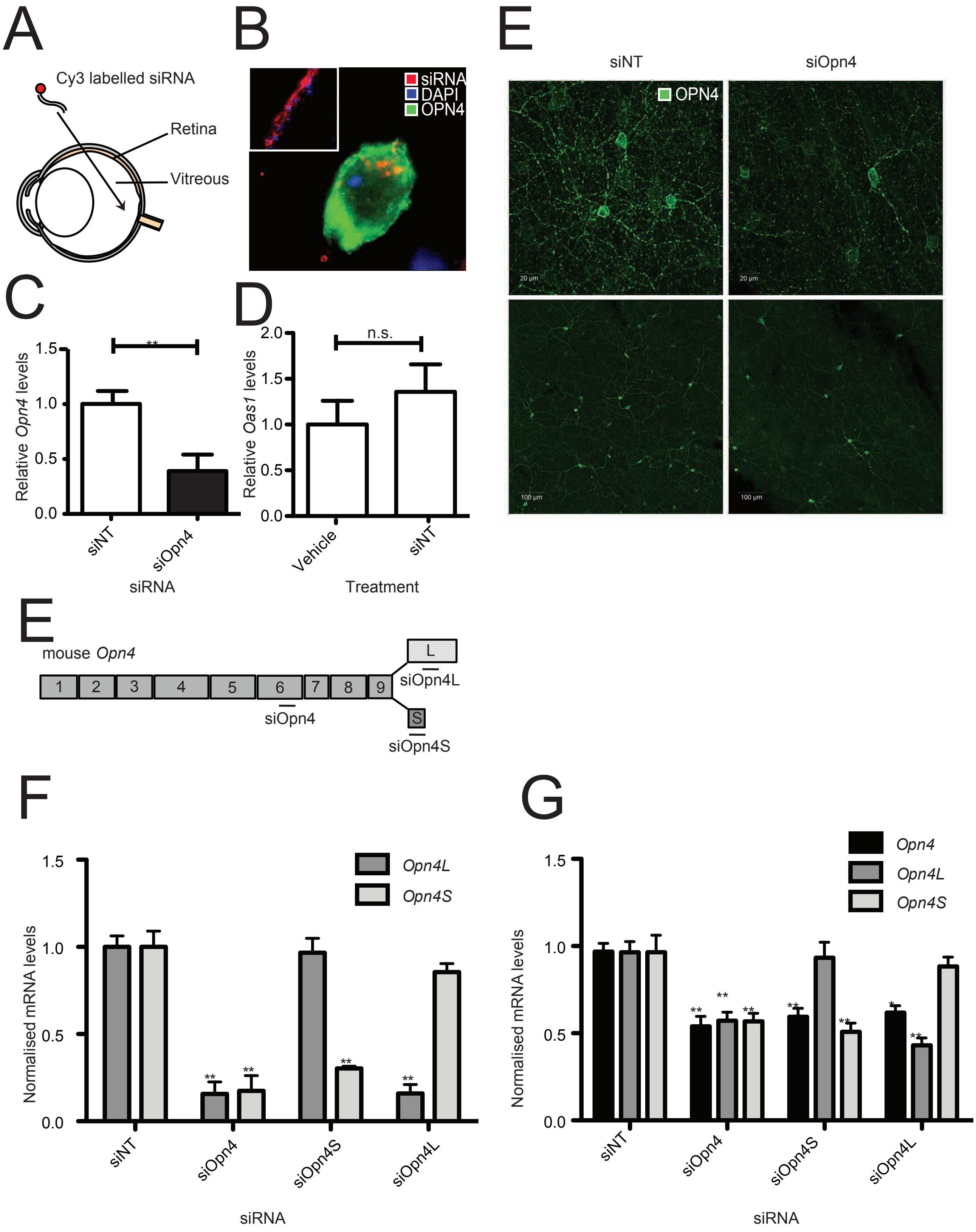

A
Cy3 labelled siRNA
Retina
Vitreous
B
siRNA
DAPI
OPN4
E
siNT
siOpn4
OPN4
C
Relative Opn4 levels
siNT
siOpn4
siRNA
D
n.s.
Relative Oas1 levels
Vehicle
siNT
Treatment
E
mouse Opn4
siOpn4
siOpn4L
siOpn4S
F
Opn4L
Opn4S
Normalised mRNA levels
siNT
siOpn4
siOpn4S
siOpn4L
siRNA
G
Opn4
Opn4L
Opn4S
Normalised mRNA levels
siNT
siOpn4
siOpn4S
siOpn4L
siRNA

Figure S3

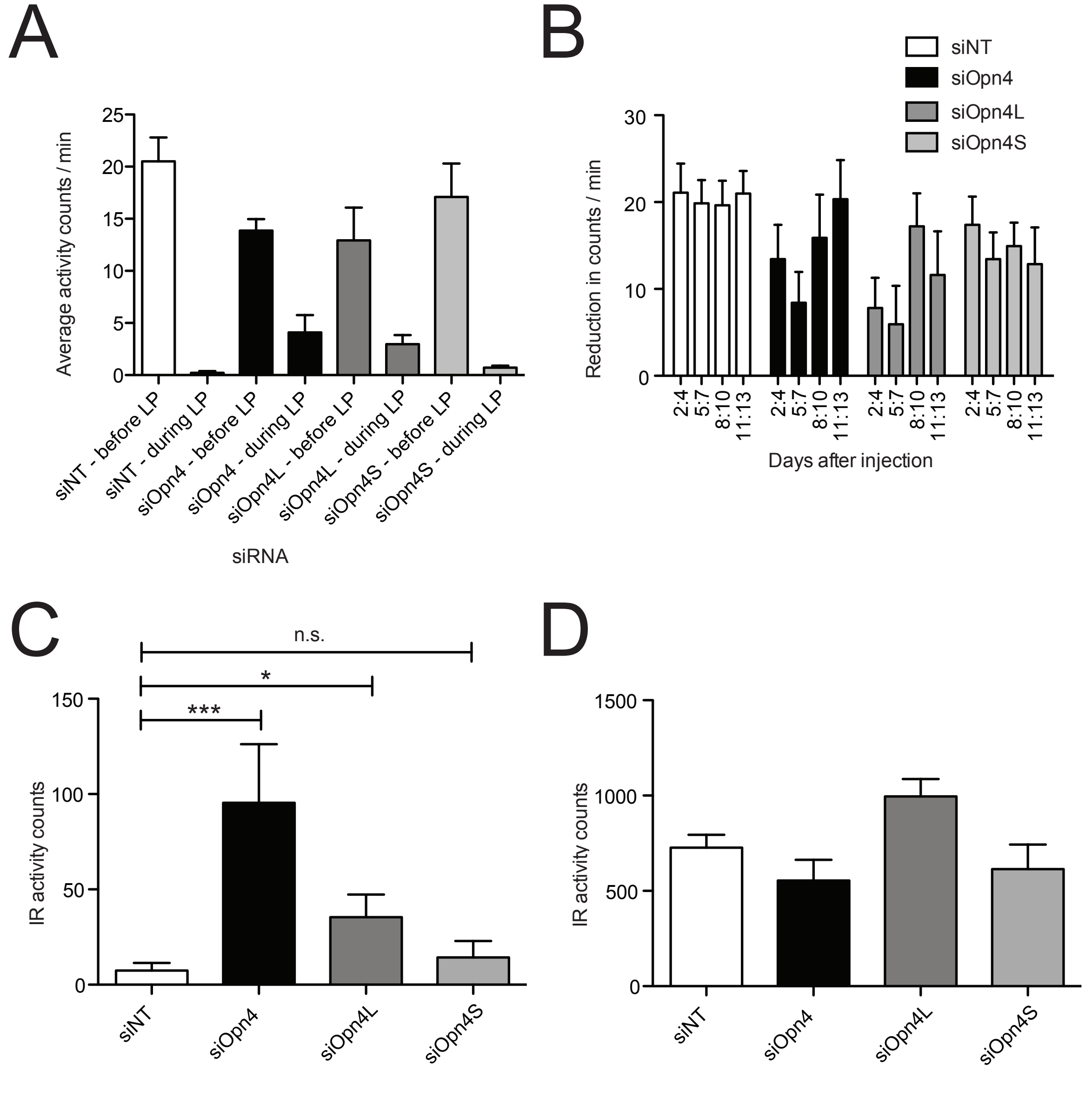

A
B
siNT
siOpn4
siOpn4L
siOpn4S
Average activity counts / min
25
20
15
10
5
0
siNT - before LP
siNT - during LP
siOpn4 - before LP
siOpn4 - during LP
siOpn4L - before LP
siOpn4L - during LP
siOpn4S - before LP
siOpn4S - during LP
siRNA
Reduction in counts / min
30
20
10
0
2:4
5:7
8:10
11:13
Days after injection
C
D
n.s.
*
***
IR activity counts
150
100
50
0
siNT
siOpn4
siOpn4L
siOpn4S
IR activity counts
1500
1000
500
0
siNT
siOpn4
siOpn4L
siOpn4S

Figure S4

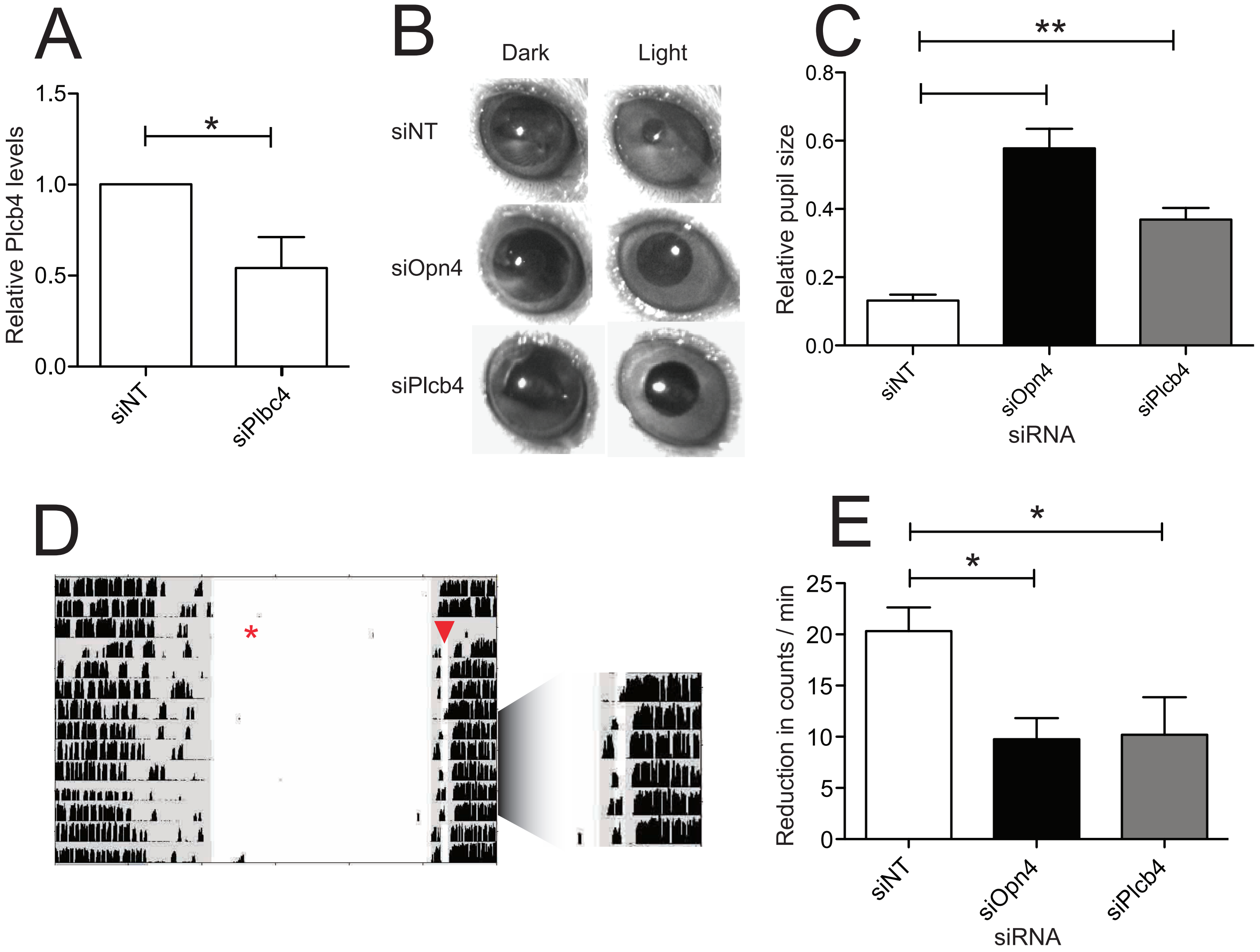
A
B
C
D
E
Relative Plcb4 levels
1.5
1.0
0.5
0.0
*
siNT
siPlbc4
Dark
Light
siNT
siOpn4
siPlcb4
Relative pupil size
0.8
0.6
0.4
0.2
0.0
**
siNT
siOpn4
siPlcb4
siRNA
Reduction in counts / min
25
20
15
10
5
0
*
*
siNT
siOpn4
siPlcb4
siRNA

Supplemental Figure Legends

Figure S1: Evidence for human *Opn4L* relating to Figs. 1-4.

A) Amplification of a partial fragment of human OPN4 long from retinal cDNA using primers HOPN47F and HOPN4Long. 1kb Ladder (Invitrogen). **B)** Nucleotide sequence and deduced amino acid sequence of 656bp PCR product generated using primers HOPN47F and HOPN4Long (sequences are shaded) from human retinal cDNA. The intron- exon boundaries are marked with vertical blue lines. The product spans exons 7, 8 and 9 and reads through the predicted end of exon 9. **C)** Clustal W alignment of the predicted amino acid sequences of human and mouse long and short isoforms. Sequences are shown from the start of primer HOPN47F. The long isoforms show 70% similarity over this sequence and the short isoforms 84% similarity.

Figure S2: siRNA-mediated silencing of *Opn4* relating to Figs. 1-4.

A) Schematic showing intravitreal injection of chemically stabilized Cy3-labelled siRNA. **B)** siRNA successfully reached the ganglion cell layer (inset), and was identified within melanopsin-expressing photosensitive retinal ganglion cells, OPN4 immunostained in green, siRNA in red and nuclei with DAPI in blue. **C)** qPCR for *Opn4* mRNA showing significant silencing in the animals treated with siRNA against *Opn4* (siOpn4) vs non-targeting siRNA (siNT) (55%, p=0.006, n=10). **D)** qPCR for *Oas1* shows no significant induction of this gene in siRNA treated retina (siNT) vs vehicle treated retina (vehicle), showing the absence of an interferon response; one of the prime concerns *in vivo* RNAi [29,30], n=6 for each group, *=p<0.05, **=p<0.01 **E)** Whole retinal flat-mounts showing markedly lower levels of OPN4 (green) in siOPN4 transfected retina. **F)** Schematic of the *Opn4* gene, showing the target position of the siRNAs against both *Opn4* isoforms (siOpn4), *Opn4L* (siOpn4L) and *Opn4S* (siOpn4S). siOpn4 matches an area in exon6, siOpn4L a region of exon 9 specific to *Opn4L* and siOpn4S exon 10, specific to *Opn4S*. **G)** qPCR of *Opn4L* and *Opn4S* from stably transfected Neuro2A cell lines expressing each isoform, treated with siRNA as indicated, showing specific silencing of each isoform by the respective siRNA. N=6, **=p<0.01, T-test. **H)** Similar results are seen *in vivo; Opn4* levels are measured with 3 primers, the

first targeting a fragment in the 5' region, thereby covering both isoforms (*Opn4*, white), the second targeting *Opn4S* (*Opn4S*, light grey), the third targeting *Opn4L* (*Opn4L*, dark grey). N=16, **=p<0.01, *=p<0.05, ANOVA with post-hoc Dunnett's tests.

Figure S3: Wheel running activity during light pulse relating to Fig. 3 and activity measurements to Fig. 4

A) Histogram of total activity before and during the 10 minute light pulse averaged through days 2-8 after injection for the four different siRNA treatments. **B)** reduction in activity during the light pulse from an equivalent period just before the pulse across 3 day bins for the four siRNAs as above. **C)** Locomotor activity as measured by PIR during the first 10 minutes of the light pulse, to compare with Fig. 3 *Opn4* and *Opn4L* knockdown significantly reduce masking. **D)** Activity as measured by PIR during the hour preceding the light pulse shows no significant differences (n=4, p=0.1, one way ANOVA) across the groups, showing sleep pressure is not a contributing factor to levels of sleep induction.

Figure S4: PLCB4 participates in OPN4L and OPN4S signalling cascades relating to Figs. 1 and 3.

A) Silencing of *Plcb4* mRNA *in vivo* (54% of control, p=0.049, n=5). **B)** Pupil images before and immediately after a 10s exposure to bright 480nm light, showing reduced pupil constriction with siOpn4 and siPlcb4. **C)** Histogram of average pupil constriction at the end of the light pulse for animals injected with siRNA as indicated, showing significantly attenuated pupil constriction for siPlcb4 (0.37 ± 0.03 n=12, p<0.0001 vs siNT). siOpn4 values provided for comparison **D)** Representative actogram from an animal given intravitreal bilateral injection (indicated by a red star) of siPlcb4 showing low levels of negative masking during a 10 min ZT12.5 light pulse (indicated by a red arrow) every day following injection. Actograms enlarged around light pulse for clarity. **E)** Histogram of reduction in wheel running activity during the nocturnal 10 min light pulse shows severely attenuated masking with siPlcb4 (83.53±12.76 n=6 vs 30.42±5.89 n=10 siNT, p=0.001) and siOpn4 values provided for comparison. *=p<0.05, **=p<0.01

Supplemental Experimental Procedures

Animals: All animals used were retinal degenerate *rd/rd* (C3H/HeN, Harlan UK) mice (older than 80 days) lacking rod and the majority of cone photoreceptors, unless otherwise indicated as *rd/rd cl* [S1]. All animals were housed under a 12:12 LD cycle with food and water ad libitum. All procedures were conducted in accordance with the Animals (Scientific Procedures) Act 1986 and the University of Oxford Policy on the Use of Animals in Scientific Research (PPL 70/6382 and 30/2812). All procedures were reviewed by the Clinical Medicine Animal Welfare and Ethical Review Body (AWERB). Animals were sacrificed via Schedule 1 methods in accordance with the UK Home Office Animals (Scientific Procedures) Act 1986.

Behavioural tests:

Pupillometry: Assessment of pupillary light responses was performed 72 hours post intravitreal injection of siRNA, using methods reported previously [S2]. Testing was performed between ZT 4-8, and all animals were dark adapted for 1–2 hours prior to testing. A xenon arc lamp (150W solar simulator, Lot Oriel, UK) with a 480 nm monochromatic filter (Andover, 10 nm half-bandwidth) was used to produce a light intensity of 14.6 log quanta/cm^2/s (173 μW/cm^2/s). Light stimuli (10 seconds) were transmitted to the eye via a liquid light pipe as an irradiant light stimulus using a 2” integrating sphere (Pro-lite Technology, UK) and was controlled by a shutter positioned in the light path (LSZ160 shutter, Lot Oriel UK; custom software supplied by BRSL, Newbury, UK). Images of consensual pupil responses were collected with a Prosilica NIR sensitive CCD video camera (BRSL, Newbury, UK) at a rate of 10 frames per second, under infrared LED illumination (850nm, 10nm half-bandwidth). During pupil measurements unanaesthetised animals were temporarily restrained using normal husbandry techniques for the duration of the recording (29 seconds, including baseline, stimulation and recovery phases). Each animal was tested 5 times to minimise any artefacts due to handling. Data reported for each individual animal represents the mean of all

individual trials. All images were analysed using ImageJ software (NIH; rsbweb.nih.gov/ij/).

Phase Shifting: Mice were maintained on running wheels in light tight chambers on a 12:12 LD cycle (100 lux from white LED lamps) and received a bilateral injection of the indicated siRNA. 3 days after the injection, the mice were placed in DD for 24 hours and received a 30 min light pulse at approximately CT16. The mice were then allowed to free run for 14 days or longer in DD, running wheel activity data were collected and analysed on Clocklab (Actimetrics, Wilmette, IL).

Masking: Mice were housed under a 12:12 LD cycle (400 lux from SpectraNova halogen white light (Schott AG, Mainz, Germany) in light tight chambers and received a bilateral injection of the indicated siRNA. The day following the injection onwards, the mice also received a 10 min light pulse at ZT12.5 every day for the following 20 days. Activity data were collected and analysed on Clocklab.

Sleep: Mice were housed under a 12:12 LD cycle (400 lux from SpectraNova halogen white light (Schott AG, Mainz, Germany) in light tight chambers and received a bilateral injection of the indicated siRNA. 4 days later, the mice were given a 1 hour light pulse at ZT14 during which videos of each mouse were recorded. Sleep was then analysed from the videos as bouts of immobility >40s [S3]. Simultaneous activity recordings were made using passive infrared monitors. Activity patterns within the cage were analysed using Anymaze (www.anymaze.com).

***In vitro* RNAi and siRNA sequences:** We used chemically stabilised siRNAs previously shown to be suitable for in vivo use [S4, S5]. siRNA sequences against *Opn4* were purchased predesigned from Invitrogen as Stealth siRNAs. siOpn4L and siOpn4S were designed by hand and ordered from Dharmacon. Silencing efficiency was tested in vitro using Neuro2A cells stably expressing either *Opn4S* or *Opn4L* as previously described [S6]. siRNAs were transfected at the indicated levels (between 5 and 50nM) using Lipofectamine RNAi max according to manufacturer's instructions. Knockdown was assessed after 48 hours. Non-targeting siRNA (siNT) was purchased from Dharmacon.

siOpn4: 5’ CACUGAUUGUCAUUCUUCUUU 3’
siOpn4L: 5’ GUAGCCUAAGGGGUGACCAUU 3’
siOpn4S: 5’ AGUCUGGAUCUCGGGAUGUAGUU 3’
siNT: 5’ CUUACGCUGAGUACUUCGAUU 3’
siPLCB4: 5’ GGAAGUAAUUGUCUUGAAAUU 3’

***In vivo* RNAi:** siRNA sequences validated in vitro were purchased as HPLC purified backbone modified sequences designed to be stabilized for in vivo use (siSTABLE, Dharmacon). siRNAs were complexed with Invivofectamine (Invitrogen2.0) for *in vivo* delivery according to manufacturer’s instructions. Animals were aneasthetized with ketamine:meditomidine. 3 ug of siRNA was delivered into each eye in a final volume of 1.5 ul, injected intravitreally with a Hamilton syringe (Hamilton Company) with a 34 gauge 10mm point style 2 needle.

mRNA extraction, qPCR and real-time PCR primer sequences: Total RNA was exracted from Neuro2A cells using the RNEasy mini kit (Qiagen) and from retina using the RNEasy Mini kit (following instructions for mRNA extraction), cDNA was prepared using the qScript cDNA synthesis kit (Quanta Biosciences) according to manufacturer’s instructions. Real-time PCR was performed as described previously [S7] using a SYBR green master mix (Qiagen Fast SYBR) with gene specific primers described previously [S7, S8].

Amplification of human *OPN4* long isoform

To confirm if a similar splicing mechanism is also seen in human the 3’ region of the human melanopsin gene (Genbank accession number AF147788) was analysed and a forward primer was designed in Exon 7 (H*OPN4*7F 5’-GCCAAGGCCTCTGCAATCCA-3’) and a reverse primer designed to the 3’ untranslated region of the predicted long isoform (H*OPN4*Long 5’-CATGGTGCTGTTGGCTGTGC-3’). PCR was performed using Platinum *Taq* Supermix (Invitrogen) with an initial denaturation step at 94°C for 3 min, then 94°C for 30 s, 57°C for 30 s, and 72°C for 1 min 30 s for 35 cycles, followed by a final extension at 72°C for 7 min. PCR was carried out using human retinal cDNA (Clontech) as template with Platinum PCR Supermix (Invitrogen)

and each 25µl reaction contained 0.2µM each primer and 0.5µl of cDNA (0.5ng). The 656bp fragment obtained was cloned into pGEM-T Easy and sequenced.

Immunohistochemistry:

Immunostaining of retina cryostat sections and whole retina flatmounts was performed as described previously [S2, S9]. Briefly, retinal sections were permeabilised in PBS with 0.2% Triton X at RT for 20 min and blocked in PBS with 10% normal donkey serum (Sigma) with 0.2% Triton X for 1 hour at RT. Rabbit polyclonal anti-melanopsin antibody recognising the N-terminus of murine Opn4common to both Opn4L and Opn4S (UF006, Advanced Targeting Systems) was diluted 1:2500 and incubated for 16 hours at 4°C diluted in 2.5% donkey serum in PBS with 0.2% Triton X. Donkey anti-rabbit Alexa-488 and Alexa-568 secondary antibodies (Life Technologies) were incubated for 2 hours at RT diluted 1:200 in 2.5% donkey serum in PBS with 0.2% Triton X. All wash steps were performed using PBS with 0.05% Tween-20. Sections were mounted in Prolong Gold anti-fade reagent containing DAPI (Life Technologies). Similar methods were employed for whole retina staining, although primary antibody was incubated for 3 days and levels of Triton X were increased to 1% for all steps. Fluorescent images were acquired using an inverted LSM 710 laser scanning confocal microscope (Zeiss) with Zen 2010 image acquisition software (Zeiss). Excitation was 405nm, 488nm and 561nm with emissions collected between 440-480, 505-550 and 600-700nm for DAPI, green and red fluorescence respectively. For all images, global enhancements of brightness and contrast were performed using ImageJ software. For comparative analysis all images were collected and processed under identical conditions.

Statistical tests:

All data were analysed and plotted on GraphPad Prism. Statistical comparisons were made using one-way ANOVA followed by Tukey's multiple comparison tests.

References:

S1. M. S. Freedman, R. J. Lucas, B. Soni, M. von Schantz, M. Munoz, Z. David-Gray, R. Foster, Regulation of mammalian circadian behavior by non-rod, non-cone, ocular photoreceptors. *Science* **284**, 502-504 (1999)

S2. S. Hughes, C. A. Pothecary, A. Jagannath, R. G. Foster, M. W. Hankins, S. N. Peirson, Profound defects in pupillary responses to light in TRPM-channel null mice: a role for TRPM channels in non-image-forming photoreception. *Eur J Neurosci* **35**, 34-43 (2012)

S3. S. P. Fisher, S. I. Godinho, C. A. Pothecary, M. W. Hankins, R. G. Foster, S. N. Peirson, Rapid assessment of sleep-wake behavior in mice. *J Biol Rhythms* **27**, 48-58 (2012)

S4. A. L. Jackson, J. Burchard, D. Leake, A. Reynolds, J. Schelter, J. Guo, J. M. Johnson, L. Lim, J. Karpilow, K. Nichols, W. Marshall, A. Khvorova, P. S. Linsley, Position-specific chemical modification of siRNAs reduces "off-target" transcript silencing. *RNA* **12**, 1197-1205 (2006)

S5. F. Czauderna, M. Fechtner, S. Dames, H. Aygun, A. Klippel, G. J. Pronk, K. Giese, J. Kaufmann, Structural variations and stabilising modifications of synthetic siRNAs in mammalian cells. *Nucleic Acids Res* **31**, 2705-2716 (2003)

S6. S. Hughes, A. Jagannath, D. Hickey, S. Gatti, M. Wood, S. N. Peirson, R. G. Foster, M. W. Hankins, Using siRNA to define functional interactions between melanopsin and multiple G Protein partners. *Cell Mol Life Sci*, (2014)

S7. A. Jagannath, R. Butler, S. I. Godinho, Y. Couch, L. A. Brown, S. R. Vasudevan, K. C. Flanagan, D. Anthony, G. C. Churchill, M. J. Wood, G. Steiner, M. Ebeling, M. Hossbach, J. G. Wettstein, G. E. Duffield, S. Gatti, M. W. Hankins, R. G. Foster, S. N. Peirson, The CRTC1-SIK1 pathway regulates entrainment of the circadian clock. *Cell* **154**, 1100-1111 (2013)

S8. S. S. Pires, S. Hughes, M. Turton, Z. Melyan, S. N. Peirson, L. Zheng, M. Kosmaoglou, J. Bellingham, M. E. Cheetham, R. J. Lucas, R. G. Foster, M. W. Hankins, S. Halford, Differential expression of two

distinct functional isoforms of melanopsin (Opn4) in the mammalian retina. *J Neurosci* **29**, 12332-12342 (2009)

S9. S. Hughes, T. S. Watson, S. N. Peirson, R. G. Foster, M. W. Hankins, Non-uniform distribution and spectral tuning of photosensitive retinal ganglion cells of the mouse retina. *Curr Biol* **Under Review**, (2013)
